# *C. elegans* Presenilin Mediates Inter-Organelle Contacts and Communication that Is Required for Lysosome Activity

**DOI:** 10.14336/AD.2024.0228

**Published:** 2025-02-28

**Authors:** Kerry C. Ryan, Zahra Ashkavand, Jocelyn T. Laboy, Ling Wang, Margarida Barroso, Kenneth R. Norman

**Affiliations:** ^1^Department of Regenerative and Cancer Cell Biology,; ^2^Department of Molecular Cellular Physiology, Albany Medical College, Albany, New York 12208 USA

**Keywords:** C. elegans, Mitochondria, Lysosomes, Calcium, Presenilin

## Abstract

Compromised lysosome function is implicated in the pathology of many neurodegenerative diseases, including Alzheimer’s disease (AD). Familial Alzheimer’s disease (fAD) is caused primarily by mutations in the presenilin encoding genes, but the underlying mechanism remains obscure. Loss of the conserved *C. elegans* presenilin orthologue SEL-12 results in increased mitochondrial calcium, which promotes neurodegeneration. Here, we find that *sel-12* mutant lysosomes, independent of SEL-12 proteolytic activity, are significantly enlarged and more alkaline due to increased ER-to-mitochondrial calcium signaling and concomitant mitochondrial oxidative stress. These defects and their dependence on mitochondrial calcium are recapitulated in human fAD fibroblasts, demonstrating a conserved role for mitochondrial calcium in presenilin-mediated lysosome dysfunction. *sel-12* mutants also have increased contact surface area between the ER, mitochondria, and lysosomes, suggesting *sel-12* has an additional role in modulating organelle contact and communication. Overall, we demonstrate that SEL-12 maintains lysosome acidity and lysosome health by controlling ER-to-mitochondrial calcium signaling.

## INTRODUCTION

The autophagy-lysosome system is a major pathway for degrading and recycling misfolded proteins and damaged organelles. Neurons rely on the autophagy-lysosome system for the preservation of protein homeostasis (proteostasis) [1, 2]. In fact, most neurodegenerative diseases are characterized by protein misfolding and aggregation indicative of a loss of proteostasis and defects in proteostasis machinery. Many studies in Alzheimer’s disease (AD) neurons and AD models show disrupted autophagy, either due to deficient autophagy induction or in defective clearance of autophagosomes by the lysosomes [3, 4]. Failure to successfully clear degradative contents has severe consequences, including Aβ and tau aggregation and cell death [5-7].

Familial Alzheimer’s disease (fAD) is predominantly caused by mutations in the presenilin-encoding genes. Presenilins are ubiquitously expressed proteins that are phylogenetically conserved and are found in multicellular organisms, including plants, invertebrates and mammals. Additionally, presenilins are largely found on endomembrane structures and form the catalytic core of the gamma secretase complex. Presenilin is linked to AD through its ability to cleave the amyloid precursor protein to generate Abeta peptides. However, presenilin also plays important roles independent of its protease activity, including in intracellular calcium flux, lipid metabolism, and autophagy [8-15]. Indeed, fAD presenilin mutations are associated with autophagy defects at multiple stages in the process including initiation, autophagosome maturation, autophagosome fusion with lysosomes, and cargo degradation by the lysosomes [16]. Consistently, we have reported that loss of the *C. elegans* presenilin 1 orthologue SEL-12 results in a loss of proteostasis [17] and disrupts autophagy [18]. In addition, we found that the proteostasis defects observed in *sel-12* mutants were dependent upon an aberrant elevation in mitochondrial calcium levels [17, 18]. There is also evidence that presenilin is necessary for proper lysosome function [10, 11, 19-22]. Lysosome morphological abnormalities are present both in presenilin fAD models and in biopsy specimens taken from AD patients [20, 23-25], suggesting lysosome dysfunction is a common feature of AD and a central aspect of AD pathology requiring further study.

Here, due to its genetic amenability and optical clarity, we use the simple in vivo model organism *C. elegans* to identify lysosomal defects in *sel-12* mutants and implicate *sel-12*-mediated calcium mitochondrial homeostasis in lysosome function. We find that the lysosomes in *sel-12* null mutants are significantly enlarged and more alkaline compared to wild type animals. Furthermore, in both *sel-12* mutants and fAD patient cells, lysosomal defects arise from elevated ER-to-mitochondria calcium signaling. Additionally, we report that loss of SEL-12 results in increased inter-organelle contacts between the ER, mitochondria, and the lysosome, suggesting that SEL-12 plays a role in regulating inter-organelle communication. Overall, these data highlight the interdependent nature of organelles and the critical role of presenilin in regulating inter-organelle contact and communication that is important for mitochondrial calcium homeostasis and lysosome function.

## MATERIALS AND METHODS

### *C. elegans* maintenance

All *C. elegans* strains were grown at 20°C on nematode growth media (NGM) plates seeded with *E. coli* OP50 unless otherwise indicated. All animals were age synchronized by bleaching gravid worms to obtain the eggs, which were then incubated in M9 for 24-48 h before being allowed to hatch. Afterward, L1 larvae were grown to adulthood on NGM plates for further experiments. Day 1 adults were analyzed for all experiments unless otherwise indicated. Genotypes were determined by PCR and DNA sequencing. The following strains were used in the study: N2 Bristol wild type, *sel-12(ar131, ok2078, tak-10, ty11*) X, *mcu-1(tm6026*) IV, *jsIs609* [*mec-4p*::MLS::GFP], *juSi271* I (*col-19p*::mito::dendra2), *pwSi82* (*phyp-7*-VITss::oxGFP-KDEL), *qxIs257* (*ced-1p*::*nuc-1*::Cherry), *qxIs750* (*hsp*::*nuc-1*::pHTomato), *takEx541 (sel-12p::sel-12*::GFP), *takEx699* (*mec-7p*::*lmp-1*::wrmScarlet), *takEx767* (*semo-1p*::2xMLS:: GCaMP6f::SL2::wrmScarlet), *takEx770* (*semo-1p*::*hsp-4ss*::wrmScarlet::KDEL), *hpEx2216* (*rgef-1p::*mCherry:: SP12), and *zdIs5* [*mec-4p*::GFP].

### Cell lines

FAD (AG06840 and AG08170) and control (AG07621) human skin fibroblast cell lines were obtained from NIA Aging Cell Culture Repository (Coriell, Camden, NJ). AmpFLSTR Indentifiler Plus PCR Amplification Kit (ThermoFisher Scientific Cat#4427368) was used to authenticate the cell lines and MycoSEQ Mycoplasm Detection System (Life Technologies Cat#NC1980761) by Coriell Cell Repositories was used to test absence of mycoplasma. Cell culture media and reagents were purchased from Invitrogen (Waltham, MA) and Corning (Cellgro, Manassas, VA). All cell lines were grown in Dulbecco’s modified Eagle’s medium (DMEM)(Cat# 11965092) at 37°C under humidified air containing 5% CO2. The DMEM (4.5 mg/L glucose, 110 mg/L sodium pyruvate) was supplemented with penicillin (100 U/ml) (Cat#15240062), streptomycin (100 μg/ml), and 15% fetal bovine serum. Cells were cultured according to the protocol provided by the cell supplier.

### DNA constructs and transgenesis

To create the *semo-1p::secretory sequence (ss): :wrmScarlet*::KDEL (*takEx770*) construct, 3 kb of the 5’ untranslated region of *semo-1* and ss::wrmScarlet::KDEL were synthesized by GenScript and combined using Gibson assembly (NEB Cat# E5510). For *takEx767* (*semo-1p*::2xMLS::GCaMP6f::SL2::wrmScarlet), the *semo-1* promoter and wrmScarlet were combined with 2xMLS::GCaMP6f::SL2 (amplified from pJL73) using Gibson Assembly (NEB). For *takEx699* (*mec-7p*::*lmp-1*::wrmScarlet), the *lmp-1* cDNA and wrmScarlet were synthesized by GenScript and the *mec-7* promoter was obtained from pPD46.41 (Fire Lab Vector Kit) and were combined using Gibson Assembly (NEB).

In brief, primers were designed to amplify the 5’ end containing the adjacent DNA fragment and the fragment at the 3’ end that will anneal to the target sequence. This creates overlapped regions at the 5’ end of the primers. The Gibson Assembly reaction contains an exonuclease that creates single-strand overhang regions that will then anneal with the adjacent DNA fragment. After ligation, the product was transformed in MAX Efficiency DH5 alpha competent cells (Invitrogen), and the final construct was verified by DNA sequencing. The constructs were injected into N2 wild-type worms using standard procedures [26], along with a *ttx-3p*::GFP co-injection fluorescent marker that labels the bilaterally symmetrical AIY neurons (gift from O. Hobert, Columbia University). Fluorescent microscopy was used to confirm the correct subcellular localization of all constructs.

### RNAi treatments

RNAi was delivered by feeding as described [27]. L1 animals were grown to day 1 of adulthood on NGM plates seeded with HT115 *E. coli* bacteria that produced either empty feeding vector, or *itr-1* or *unc-68* double-stranded RNA. These bacteria were obtained from the Ahringer library [28] and production of *itr-1* and *unc-68* was verified by PCR. Day 1 adult animals were analyzed.

### Calcium imaging

Mitochondrial calcium concentration was measured in the *C. elegans* hypodermis in animals expressing *semo-1p*::2xMLS::GCaMP6f::SL2::wrmScarlet. Animals were first immobilized on slides using 500mM levamisole on 2% agarose pads. Images of the animals were captured using a 63x objective lens on a Zeiss Axio Observer microscope equipped with an Andor Clara CCD camera. The ratio of GCamP6f::SL2::wrmScarlet fluorescence intensity was quantified so that GCaMP6f fluorescence was normalized to wrmScarlet intensity. Metamorph software was used to compile images.

### Drug treatments

N-acetyl cysteine (NAC) (Sigma-Aldrich, Cat#A7250) was prepared in DMSO and added to NGM plate media at 9 mM, and animals were moved to NAC plates as L4 animals until adulthood, as previously described [17]. For mitoTEMPO experiments, either 500 μM of (2-(2,2,6,6-tetramethylpiperidin-1-oxyl-4-ylamino) -2-oxoethyl) triphenylphosphonium chloride (mitoTEMPO) (Sigma-Aldrich Cat# SML0737) or 500 μM triphenyl-phosphonium chloride (TPP) (Sigma-Aldrich Cat# 11726LE) was added to NGM plates. Animals were moved to mitoTEMPO or TPP plates as L1 larvae.

### Quantification of lysosome volume and acidity

Lysosomes were imaged in transgenic animals expressing NUC-1::CHERRY driven by the ubiquitous *ced-1* promoter (*qxIs257*). These animals were co-expressing *mec-4p*::GFP (*zdIs5*) to mark the touch receptor neurons; lysosomes present within the GFP signal were identified as touch receptor neuron lysosomes. All images were captured at 60× magnification using a Nikon laser-scanning confocal microscope with NIS Elements software, with z-stacks acquired at 0.25 µm slice intervals. For hypodermis lysosomes, a selection of lysosomes at the midbody of the worm was analyzed and lysosome volume was averaged for each worm. Volume was quantified using Imaris 9.0 software. 20 animals per strain were analyzed.

To compare the relative pH between strains, we examined animals expressing NUC-1::pHTomato, where pHTomato is a pH-sensitive fluorescent protein. This construct is driven under the heat shock promoter. To induce expression, animals were heat shocked at 37°C for 30 minutes, then incubated at 20°C for 24 hours so that the transgenic protein could enter the lysosome [29]. Images of the lysosomes in the hypodermis were taken at 60× magnification using a Nikon laser-scanning confocal microscope with NIS Elements software, with z stacks acquired at 0.25 µm slice intervals. All settings for pinhole size and laser power were identical for all images taken. Imaris 9.0 software was used to quantify the mean fluorescent intensity of each lysosome. 20 animals per strain were analyzed.

### Lysosome volume measurements in FAD fibroblasts

To image fibroblast lysosomes, live cells were plated onto 35mm glass bottom dishes (MatTek Corp) and left to attach overnight. The following day, the cells were either incubated for 1 hour with 100nM LysoTracker red DND99 dye (Invitrogen Cat# L7528) or labeled with Celllight™ Lysosomes RFP BacMam 2.0 (Invitrogen Cat# C10597) overnight at 37°C. Cells were imaged immediately following incubation using confocal microscopy. For Ru265 treatment, fibroblasts were treated with 10 μM Ru265 (Calbiochem) for 48 hr. Imaris 9.0 software was used to quantify lysosome volume, and at least 20 cells were imaged per line.

### Inter-organelle contact surface area

To measure mitochondria-lysosome contacts within the hypodermis, transgenic animals were generated co-expressing *qxIs257* (*ced-1p*::NUC-1::Cherry) to mark the lysosomes and *juSi271* I (*col-19p*::mito::dendra2) to mark the mitochondria. Similarly, lysosome-ER contacts were measured in animals co-expressing an ER marker [*pw*Si82 (phyp-7-VITss::oxGFP-KDEL)] and lysosome marker (*qxIs257*), and ER-mitochondria contacts were measured in animals co-expressing *juSi271* and *takEx770 (semo-1p::wrmScarlet::KDEL).* Animals were immobilized on slides using 500mM levamisole on 2% agarose pads. All images were captured at 60× magnification using a Nikon laser-scanning confocal microscope with NIS Elements software, with z-stacks acquired at 0.25 µm slice intervals through the depth of the hypodermis. Images were taken at the midbody of each animal. Colocalization analysis was performed on Imaris 9.0 using the XTension “Surface Contact Area” Imaris plugin (https://imaris.oxinst.com/open/view/surface-surfacecontact-area), as described previously [30], with the “surface” module used to create a 3D rendering of organelle surfaces, and background subtraction held constant within an experiment. The ratio of contact surface area: total surface area was determined by summing the contact area within the field of view and dividing by the area of the hypodermis within the field of view. 20 animals per strain were analyzed.

### Quantification and statistical analysis

Statistical difference comparing two treatment groups was determined using a Student's t test, and for parametric data, a one-way analysis of variance with a Tukey post hoc analysis was used for multiple comparisons. Normal distribution of these data was determined using the Shapiro-Wilk test. For non-parametric data, Kruskal-Wallis test with Dunn’s post hoc analysis was used. For the coelomocyte morphology analysis, a chi-square test was used to determine statistical difference between genotypes. A p value of less than 0.05 was considered significant. Graph Pad Prism software was used for all statistical analyses.

**Figure 1.**
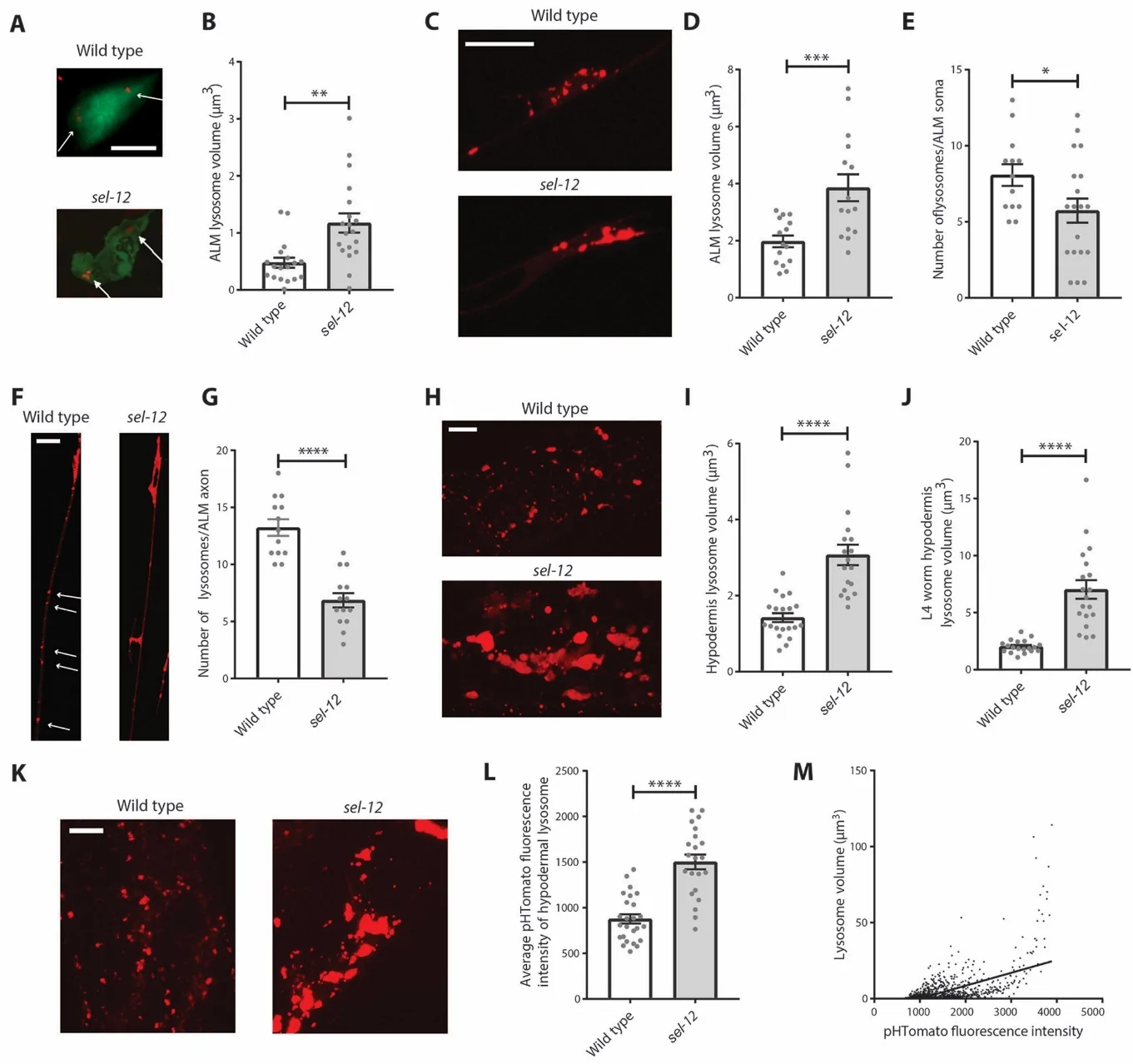
***sel-12* mutant lysosomes are enlarged and more alkaline**. (**A**) Representative images of wild type and *sel-12(ty11)* lysosomes within the ALM soma in animals co-expressing *nuc-1*::mCherry as a marker for lysosomes (arrows) and *mec-4p*::GFP to mark the TRNs (scale bar = 5 µm). (**B**) Quantification of average lysosome volume within the ALM TRN soma in wild type and *sel-12* animals (n ≥ 18 animals). (C and D) Representative images of wild type and *sel-12(ty11)* lysosomes in the ALM soma in animals expressing *mec-7p::lmp-1*::wrmScarlet (C) (scale bar = 5 µm), and quantification of average lysosome volume (D) (n ≥ 18 animals). (**E**) Quantification of the number of lysosomes per soma in animals expressing *mec-7p::lmp-1*::wrmScarlet (n ≥ 14 animals). (F and G) Representative images of wild type and *sel-12* lysosomes (arrows) in the axons of animals expressing *mec-7p::lmp-1*::wrmScarlet (scale bar = 5 µm) (F) and quantification of the number of *lmp-1*::wrmScarlet puncta per ALM axon (G) (n ≥ 13). (H and I) Representative images of hypodermal lysosomes in animals expressing *nuc-1*::mCherry as a marker for the lysosomal lumen (scale bar = 10 µm) (H) and quantification of average hypodermal lysosome volume (I) (n ≥ 18 animals). (**J**) Lysosome volume (*nuc-1*::mCherry) in L4 wild type and *sel-12* animals (n ≥ 19 animals). (K and L) Representative images (K) and quantification of the average pHTomato fluorescence intensity per lysosome in animals expressing *nuc-1*::pHTomato controlled by the heat-shock promoter (L) (scale bar = 10 µm) (n ≥ 21 animals). Increased pHTomato fluorescence intensity indicates increased pH. (**M**) Linear regression for pHTomato fluorescence volume and intensity in *sel-12(ty11)* mutants (p < 0.001). ns p > 0.05, * p < 0.05, ** p < 0.01, *** p < 0.001, **** p < 0.0001. For D, G, I, J, and L, a two-tailed Student’s t test was used. For B and E, Kruskal-Wallis test was used. Error bars indicate mean ± SEM.

## RESULTS

### sel-12 mutants display abnormal lysosome morphology and impaired lysosomal function

There is evidence that presenilin plays an important role in lysosome function [22] [4, 12], but the underlying mechanism is unclear. The lysosomes in *C. elegans sel-12* mutants have not yet been characterized. Therefore, we first examined lysosome morphology and distribution in the touch receptor neurons (TRNs). The TRNs undergo stereotyped age-associated neurodegeneration [31, 32] and this occurs prematurely in *sel-12* mutants [13]. We utilized transgenic animals expressing TRN-specific soluble GFP [33] and mCherry tagged NUC-1 [29], a lysosomal nuclease and a marker for the lysosomal lumen [34]. TRN lysosomes, visualized as small puncta in day 1 adult animals, were distinguished by the colocalization of GFP and mCherry markers in neuronal cell bodies (Fig. 1A). We introduced these markers into three *sel-12* mutants: *sel-12(ty11)*, which contains a premature stop codon in *sel-12* that leads to a predicted truncated protein and null mutant [35]; *sel-12(ar131),* which contains a missense mutation that is conserved with an fAD-linked presenilin mutation [36]; and *sel-12(ok2078),* a deletion mutant [17]. We found a significant increase in lysosome volume in the *sel-12* mutant TRNs (Fig. 1A, B, S1A). As an additional strategy for visualizing TRN lysosomes, we expressed wrmScarlet [37] tagged to the lysosome-associated membrane protein 1 (LAMP1) driven under a TRN-specific promoter. Similarly, we found that while wild type animals showed small evenly sized lysosomes, the lysosomes in the *sel-12* mutants were significantly enlarged (Fig. 1C, D). We also found that *sel-12* mutants had a significant reduction in the number of lysosomes within their ALM soma (Fig. 1E) and axons (Fig. 1F, G). To examine lysosome characteristics in more detail, we looked at the lysosomes in the hypodermis; since the hypodermis is one of the largest tissues in *C. elegans,* lysosomes are easily visualized. In addition, the hypodermis, like most *C. elegans* tissues, is post-mitotic so it recapitulates the reliance of neurons upon the autophagy-lysosome system. Examining the *C. elegans* hypodermis therefore allows more detailed analyses of lysosomes in a cell that shares a sensitivity to lysosome dysfunction. To examine lysosomes in the hypodermis, we introduced a NUC-1::mCherry transgene driven under a ubiquitous promoter into the *sel-12* mutant background [29]. Consistent with our analyses of the TRNs, we found that *sel-12* animals also had enlarged lysosomes in the hypodermis (Fig. 1H, I, Supplementary Fig. 1B).

We previously found that neuronal and behavioral defects in *sel-12* mutants do not appear prior to adulthood. Indeed, *sel-12* mutants at the L4 larval stage, the stage prior to adulthood, are phenotypically wild type [13]. We examined whether the abnormal lysosome morphology precedes neuronal dysfunction and found that L4 *sel-12* mutants have enlarged lysosomes compared to L4 wild type animals (Fig. 1J). This indicates that aberrant lysosome morphology occurs prior to major signs of neurodegeneration and may indicate a causal relationship between lysosome defects and neurodegeneration. Notably, enlarged hypodermal lysosomes have been observed in aged animals [29], suggesting premature lysosomal dysfunction in *sel-12* mutants.

Next, we asked whether the lysosome morphological abnormalities indicated lysosome dysfunction. To accomplish this, we measured relative lysosome pH by examining animals transiently expressing NUC-1 fused with the pH sensitive fluorescent protein pHTomato using a heat shock inducible promoter [29]. pHTomato’s emission intensity is highly pH-dependent, and increases with higher pH [38]. By comparing pHTomato fluorescence intensity between wild type and *sel-12* mutants, we found that *sel-12* mutants had a significantly higher pHTomato fluorescence intensity, indicating that *sel-12* mutant lysosomes are more alkaline, demonstrating lysosomal dysfunction (Fig. 1K, L, S1C). Lysosome volume also correlated with pHTomato fluorescence intensity, suggesting that lysosomal enlargement is associated with impaired lysosome function (Fig. 1M). Overall, we found that *sel-12* mutants have significant lysosome morphological and functional defects, consistent with other studies on lysosomal biology in other AD and presenilin fAD models [20, 22-24, 39].

### Abnormal lysosome morphology is independent of gamma-secretase proteolytic activity in sel-12 mutants

To explore causes of lysosome enlargement, we examined whether lysosome morphology defects in *sel-12* mutants are the result of SEL-12’s proteolytic function. SEL-12, like mammalian presenilin, is the proteolytic subunit of the gamma secretase complex, which is responsible for cleaving the Notch receptor and other type I integral membrane proteins. Presenilin/gamma secretase is associated with fAD via its role in processing Abeta peptides, but there is also evidence that presenilin additionally contributes to fAD pathology through alternate gamma-secretase-independent mechanisms [8-13, 15]. To determine whether the lysosome morphology in *sel-12* mutants is impacted by *sel-12* protease activity, we examined the lysosomes in a *sel-12* CRISPR/Cas9 engineered mutant that contains a D to A mutation within the endogenous *sel-12* gene that codes for a residue necessary for its aspartyl-protease activity, D226A, and which we have previously verified abolishes gamma secretase function [17]. Hypodermal lysosome volume in *sel-12(D226A)* mutants was not significantly different from wild type animals, while it was significantly smaller compared to *sel-12* mutant lysosomes (Supplementary Fig. 1D), indicating that SEL-12’s impact on the lysosome is gamma-secretase independent.

**Figure 2.**
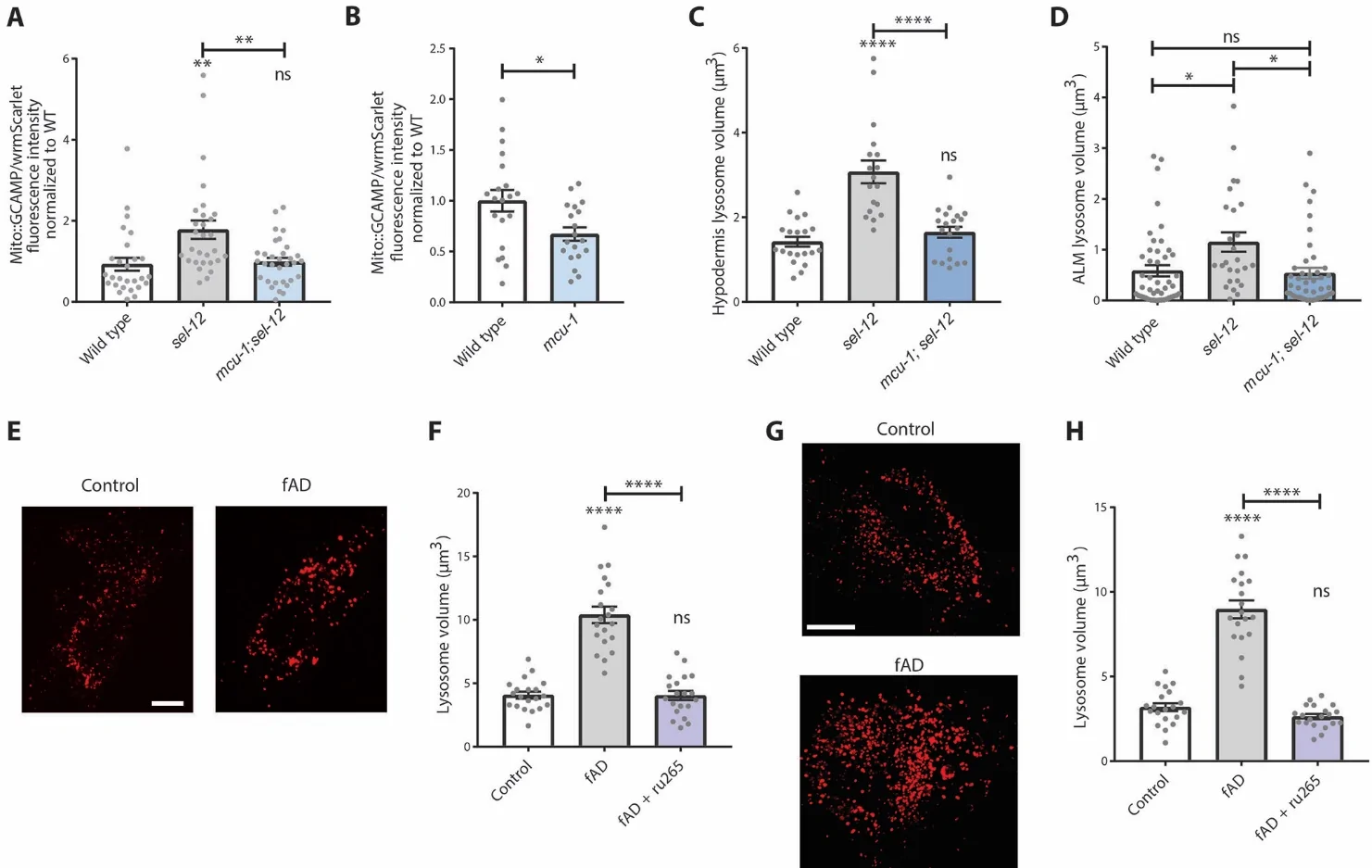
***sel-12*-mediated lysosome dysfunction is ameliorated by blocking mitochondrial calcium uptake**. (A and B) Fluorescence intensity of mitochondrial-targeted GCaMP6, a genetically encoded calcium indicator, in the hypodermis of animals also expressing cytosolic wrmScarlet as an internal expression control (*semo-1p*::2xMLS::GCaMP6f::SL2::wrmScarlet). (**C**) Quantification of hypodermal lysosome volume in wild type, *sel-12*, and *mcu-1; sel-12* animals expressing *nuc-1*::mCherry as a marker for the lysosomal lumen (n ≥ 18 animals). (**D**) Lysosome volume within the ALM soma in animals co-expressing *nuc-1*::mCherry to mark the lysosomes and *mec-4p*::GFP to mark the TRNs (n ≥ 18 animals). (E and F) Representative images of lysotracker-labeled lysosomes in fibroblasts isolated from control and fAD patients (scale bar = 15 µm) and (F) quantification of lysosome volume (+/- ru265) (n = 20 cells). (G and H) Representative images of lysosomes in control and fAD patient fibroblasts transfected with Lamp1-RFP (Celllight BacMam 2.0, Invitrogen) (scale bar = 20 µm), and (H) quantification of lysosome volume (+/- ru265) (n = 20 animals). ns p > 0.05, * p < 0.05, ** p < 0.01, *** p < 0.001, **** p < 0.0001. For B, C, F, and H, one-way ANOVA with Tukey’s multiple comparisons test was used. For A and D, Kruskal-Wallis test with Dunn’s multiple comparison test was used. Error bars indicate mean ± SEM. Comparisons are made to wild type unless otherwise indicated.

### Lysosome abnormalities are suppressed by reducing mitochondrial calcium uptake

We previously determined that aberrant mitochondrial calcium homeostasis contributes to neurodegeneration in *sel-12* mutants [13, 17]. Therefore, we wished to establish the connection between mitochondrial calcium, lysosome dysfunction, and neurodegeneration. To determine whether mitochondrial calcium causes lysosome enlargement and dysfunction, we first asked whether mitochondrial calcium is elevated in the *sel-12* hypodermis as it is in the neurons [13]. We expressed a mitochondrial targeted GCaMP calcium sensor, driven under a hypodermal-specific promoter, co-expressing a wrmScarlet marker as an expression control. We found calcium levels were elevated in hypodermal mitochondria in *sel-12* mutants (Fig. 2A). Additionally, using a mitochondrial-targeted GFP driven by a hypodermis-specific promoter, we found the hypodermal mitochondrial network is disorganized and fractured (Supplementary Fig. 2). These data are consistent with previous results in *sel-12* neurons, where elevated calcium levels correlated with mitochondrial morphological defects [13]. To confirm whether increased mitochondrial calcium uptake in the hypodermis is responsible for the fractured mitochondrial network, we suppressed mitochondrial calcium uptake in *sel-12* mutants with a mitochondrial calcium uniporter (*mcu-1*) null mutation, which we and others have shown reduces mitochondrial calcium levels (Fig. 2B)[13, 40, 41]. Introducing the *mcu-1* mutation into the *sel-12* mutant background prevented the increased mitochondrial calcium levels (Fig. 2A) and mitochondrial network fragmentation (Supplementary Fig. 2), indicating elevated mitochondrial calcium uptake in the hypodermis following SEL-12 loss causes mitochondrial disorganization and morphological changes (Supplementary Fig. 2). This also provides evidence that the hypodermis mimics the aberrant calcium signaling present in *sel-12* mutant neurons and the resulting mitochondrial abnormalities [13].

**Figure 3.**
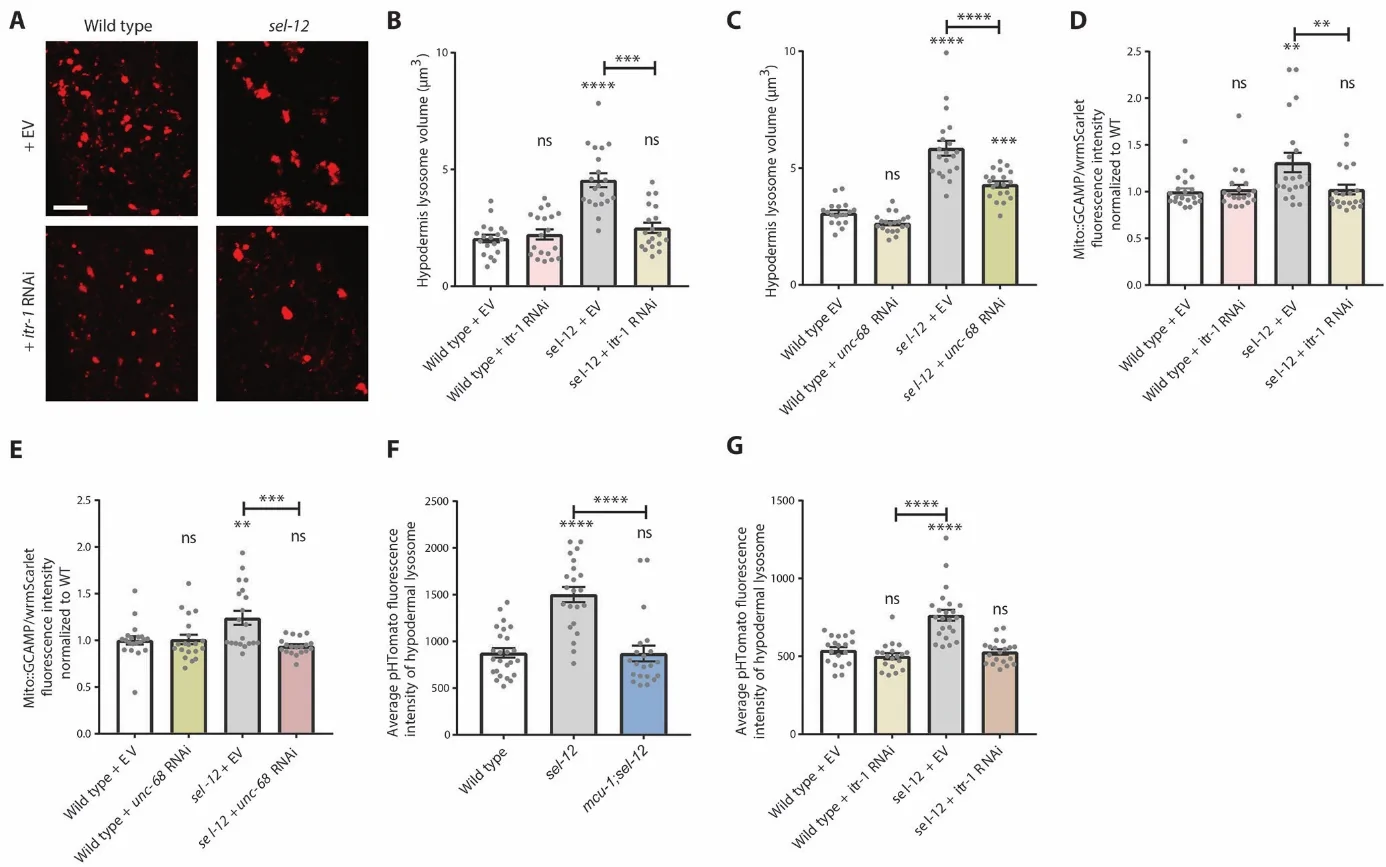
**Aberrant ER-to-mitochondrial calcium transfer results in enlarged, more alkaline lysosomes**. (A and B) Representative images of lysosomes (*nuc-1*::mCherry) in wild type and *sel-12(ty11)* animals grown on either control empty vector (EV) or *itr-1* RNAi (A) (scale bar = 10 µm), and quantification of lysosome volume (B) (n ≥ 19 animals). (**C**) Quantification of lysosome volume (*nuc-1*::mCherry) in animals grown on either EV or *unc-68* RNAi (n ≥ 18 animals). (D and E) Fluorescence intensity of hypodermal mitochondrial-targeted GCaMP6, a genetically encoded calcium indicator, normalized to cytosolic wrmScarlet, used as an internal expression control (n ≥ 19 animals). (**F**) Quantification of the average pHTomato fluorescence intensity per lysosome in animals expressing *nuc-1*::pHTomato controlled by the heat-shock promoter, with increased pHTomato fluorescence intensity indicating increased pH (n ≥ 21 animals). (**G**) *nuc-1*::pHTomato fluorescence in wild type and *sel-12(ty11)* animals grown on either EV or *itr-1* RNAi (n ≥ 19 animals). ns p > 0.05, * p < 0.05, *** p < 0.001, **** p < 0.0001. For C and G, one-way ANOVA with Tukey’s multiple comparisons test was used. For B, D, E, and F, Kruskal-Wallis test with Dunn’s multiple comparison test was used. Error bars indicate mean ± SEM. Comparisons are made to wild type unless otherwise indicated.

To determine whether this increase in mitochondrial calcium also contributes to lysosomal enlargement, we compared *sel-12* mutants with *mcu-1; sel-12* double mutants expressing the transgenic NUC-1 lysosomal marker. We found that *mcu-1; sel-12* mutants had reduced lysosome volume in both the hypodermis (Fig. 2C) and in the TRNs (Fig. 2D) and resembled wild type lysosomes, implicating increased mitochondrial calcium uptake in altered lysosome morphology.

We next asked whether the effect of mitochondrial calcium on lysosomes is conserved in human cells. Studies examining cell lines from patients that harbor fAD presenilin mutations have found enlarged lysosomes [10, 20, 24] but the cause of the enlargement remains unclear. Therefore, we first determined lysosome size in fibroblasts isolated from patients with PSEN1 fAD mutations. We have previously found evidence that the fAD fibroblasts have elevated mitochondrial calcium [13]. Using lysotracker to label live fibroblast lysosomes, we found that, like previous studies and similar to the *sel-12* mutants, fAD fibroblasts had enlarged lysosomes (Fig. 2E, F). Importantly, fAD cells treated with the mitochondrial calcium uniporter inhibitor, ruthenium 265 (ru265), showed significantly lower lysosome volume compared to untreated fAD fibroblast (Fig. 2F). Alternatively, we labeled the lysosomes by transfecting fibroblast cells with a Lamp1-RFP fusion vector. Using this method, we also found ru265 treatment rescued increased lysosome volume in fAD cells (Fig. 2G, H). Considering that previous data show mitochondrial calcium is elevated in these fAD cells [13], this suggests that this potential pathogenic mechanism is conserved in human cells.

### Altered ER-to-mitochondrial calcium signaling mediates lysosomal morphology and functional defects

Elevated ER calcium release has been implicated in neuronal dysfunction in fAD models [42-44]. Additionally, we have previously demonstrated that impairing calcium release from the ER reduces mitochondrial calcium and prevents neuronal dysfunction in *sel-12* mutants [13]. There is also evidence of increased ER-mitochondrial contacts in fAD presenilin patient cell lines [8, 45]. Therefore, we asked whether ER calcium release contributes to the lysosomal enlargement and alkalization observed in *sel-12* mutants. To inhibit ER calcium release, we treated *sel-12* mutants with RNAi knockdown of *itr-1*, which encodes the *C. elegans* inositol 1, 4, 5-trisphosphate receptor (IP3R) orthologue. Compared to *sel-12* mutants grown on control empty vector (EV) RNAi, *itr-1(RNAi); sel-12* animals had significantly reduced lysosome volume indistinguishable from those of wild type animals (Fig. 3A, B). As an alternative strategy to inhibit ER calcium release, we treated *sel-12* mutants with RNAi knockdown of *unc-68*, which encodes the *C. elegans* orthologue of the ryanodine receptor. Although not as robust as *itr-1* depletion, we found that *unc-68* RNAi treatment also reduced lysosome volume (Fig. 3C). Furthermore, both *itr-1(RNAi)* and *unc-68(RNAi)* knockdown reversed the elevation of mitochondrial calcium levels in *sel-12* mutants (Fig. 3D and E), demonstrating that blocking ER calcium release prevents excessive mitochondrial calcium uptake. Together with the reduced lysosome volume data observed in *mcu-1; sel-12* animals (Fig. 2C), these data show that calcium transfer from the ER into the mitochondria impacts lysosome morphology.

Next, we also wished to determine whether ER-to-mitochondrial calcium signaling is responsible for acidification defects found in *sel-12* mutant lysosomes. We examined *nuc-1*::pHTomato fluorescence intensity after blocking ER calcium release or mitochondrial calcium uptake in *sel-12* mutants. Either *itr-1* RNAi depletion or *mcu-1* knockout in the *sel-12* mutant background improved lysosome acidity as measured by a reduction in pHTomato fluorescence (Fig. 3F, G).

Overall, these data show that altered calcium signaling between the ER and mitochondria has morphological and functional consequences on the lysosome, an organelle critical for protein and organelle homeostasis. The data demonstrates that a defect in inter-organelle communication between the ER and mitochondrial impacts the morphology and function of the lysosome, signifying the interdependent nature of organelles and highlights that organelles do not act as solitary compartments.

### sel-12 mutants have increased mitochondria and ER contact that is dependent on inositol 1,4,5-trisphosphate receptor (ITR-1) function

fAD presenilin mutations have previously been linked with increased contact sites between the ER and the mitochondria, which increased signaling and exchange of materials between the two organelles [8, 46]. Presenilin predominantly localizes to the ER and is enriched at the region of ER membrane that is in direct contact with mitochondria [47]. In line with this, presenilin can affect ER-mitochondria calcium and lipid signaling by regulating the level of physical contact between the ER and mitochondria [8, 13, 45, 47]. It has therefore been speculated that presenilin mediates ER-mitochondrial communication, and that AD at least partially results from dysregulated inter-organelle signaling [48]. Therefore, we asked whether loss of SEL-12 recapitulated this potentially pathogenic mechanism in *sel-12* null mutants, and whether altering ER-mitochondrial calcium exchange would disrupt ER-mitochondrial contact. To first determine if SEL-12, like mammalian presenilin, localizes to the ER, we examined animals co-expressing a functional SEL-12::GFP fusion protein [13] and an ER reporter (mCherry::SP12) [49]. Similar to previous studies [50], SEL-12 showed perinuclear expression and colocalized with the ER marker, indicating SEL-12 localizes to the ER (Supplementary Fig. 3).

**Figure 4.**
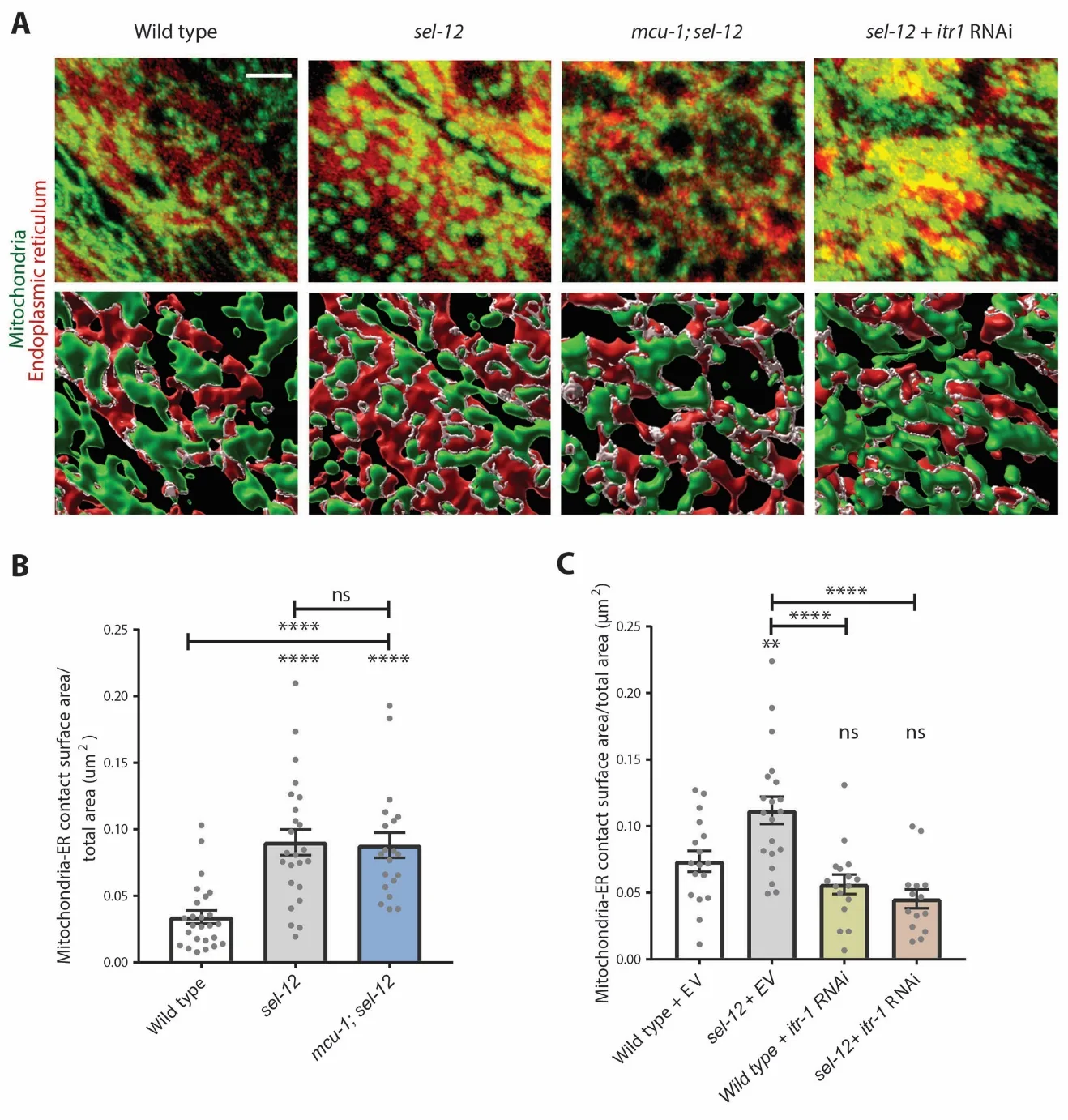
***sel-12* mutants have increased mitochondria and endoplasmic reticulum contact points dependent upon *itr-1* function but not *mcu-1***. (**A**) Representative confocal z-stack images of hypodermal mitochondria (green) and endoplasmic reticulum (red) and rendering of 3D images (bottom panels) with white indicating ER-mitochondrial contact points (scale bar = 4 µm). (**B**) Quantification of the ratio of mitochondria-ER contact surface area to total area of visible hypodermis in wild type, *sel-12(ty11)*, and *mcu-1; sel-12(ty11)* animals (n ≥ 20 animals). (**C**) Mitochondrial-ER contact surface to total surface area ratio in animals treated with either empty vector (EV) or *itr-1* RNAi (n ≥ 15 animals). ns p > 0.05, *p < 0.05, ****p < 0.0001 using Kruskal-Wallis with Dunn’s post-hoc test. Error bars indicate mean ± SEM. Comparisons are made to wild type unless otherwise indicated.

To examine contacts between the ER and mitochondria, we generated transgenic animals co-expressing an ER marker (wrmScarlet::KDEL) and a mitochondrial marker (mito::dendra2) [51]. We imaged ER-mitochondrial contacts in the hypodermis at a defined region of interest at the center of the animal, and quantified organelle contacts following a previously established methodology for 3D rendering and surface contact analysis [30]. Consistent with previous studies in presenilin fAD cell models [8, 45-47], we found that the ER-mitochondrial contact area was increased in *sel-12* mutants (Fig. 4A, B). However, inhibition of mitochondrial calcium uptake via the *mcu-1* null mutation did not alter the level of mitochondria-ER contact in *sel-12* mutants (Fig. 4A, B). This suggests that increased mitochondrial calcium is a consequence of increased ER-mitochondria contact rather than a cause. It also proposes that SEL-12 acts at the ER to influence ER-mitochondrial connections, which promotes ER-mitochondrial calcium exchange. In contrast, depleting *itr-1* via RNAi prevented the increase in ER-mitochondrial contacts in *sel-12* mutants (Fig. 4A, C). This may indicate that either *itr-1* function in ER calcium release promotes ER-mitochondria connections and/or the ITR-1 protein itself acts to mediate ER-mitochondrial contacts. Indeed, evidence in the literature shows inositol 1,4,5-trisphosphate receptor is critical for mediating ER-mitochondrial contact [52-54].

**Figure 5.**
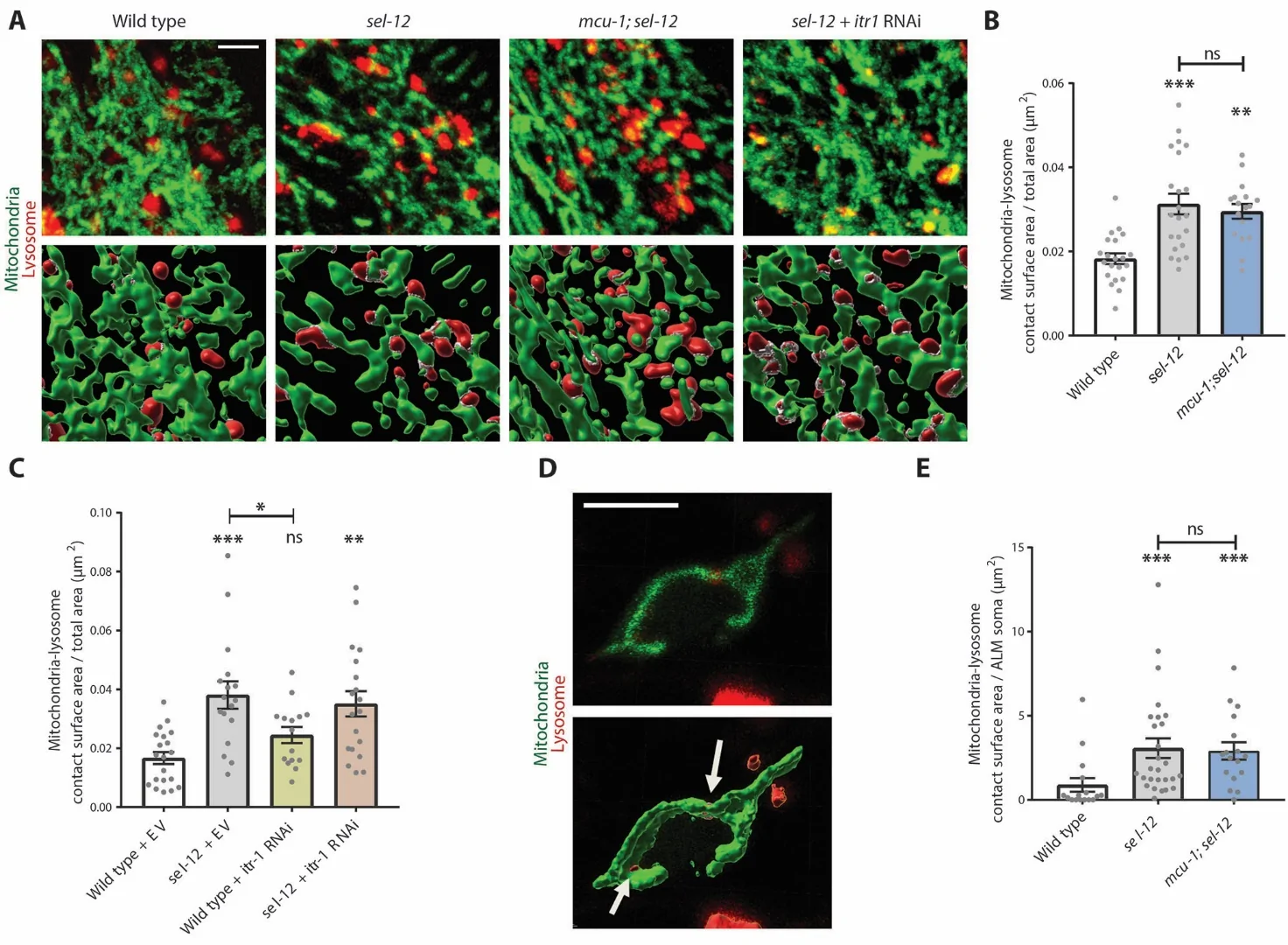
***sel-12* mutants have increased mitochondria and lysosome contact points**. (**A**) Representative confocal z-stack images of hypodermal mitochondria (green) and lysosomes (red) (scale bar = 4 µm) and rendering of 3D images (bottom panels) with white indicating mitochondria-lysosome contact points (scale bar = 4 µm). (**B**) Quantification of the ratio of mitochondria-lysosome contact surface area to total area of visible hypodermis in wild type, *sel-12(ty11)*, and *mcu-1; sel-12(ty11)* animals (n ≥ 17 animals). (**C**) Mitochondrial-lysosome contact surface to total surface area ratio in animals treated with either empty vector or *itr-1* RNAi (n ≥ 15 animals). (**D**) Representative images of confocal z-stack (upper panel) and 3D rendered (lower panel) images of ALM soma in animals co-expressing a mitochondrial marker driven under a TRN-specific promoter and a lysosome marker, with arrows indicating contact sites between lysosomes and neuronal mitochondria (scale bar = 5 µm). (**E**) Quantification of mitochondria-lysosome contact surface area per ALM soma (n ≥ 16 animals). ns p > 0.05, *p < 0.05, ** p < 0.01, ***p < 0.001. For B and C, one-way ANOVA with Tukey’s multiple comparisons test was used. For E, Kruskal-Wallis test with Dunn’s multiple comparison test was used. Comparisons are to wild type unless otherwise indicated. Error bars indicate mean ± SEM.

### Inter-organelle contacts between mitochondria-lysosomes and lysosomes-ER are increased in sel-12 mutants

Presenilin has also been found to localize to lysosomal membranes [10, 47, 55, 56]. Therefore, loss of presenilin may additionally influence communication between lysosomes and other organelles. Since we observed increased contacts between the mitochondria and ER, we asked whether other inter-organelle contacts were altered in *sel-12* mutants.

**Figure 6.**
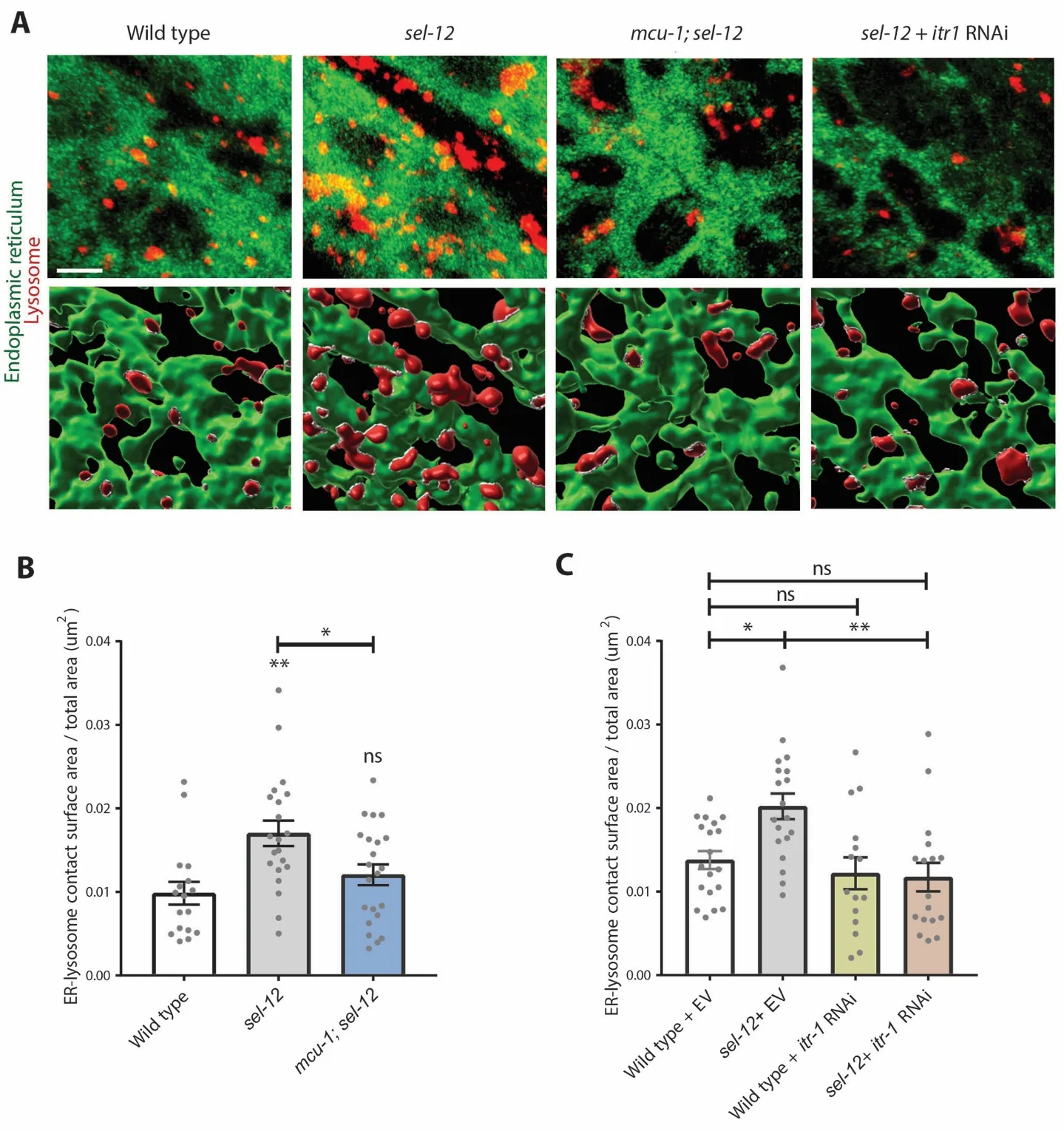
***sel-12* mutants have increased endoplasmic reticulum and lysosome contact points dependent upon ER-to-mitochondria calcium signaling**. (**A**) Representative confocal z-stack images of hypodermal endoplasmic reticulum (green) and lysosomes (red) and rendering of 3D images (bottom panels) with white indicating ER-lysosome contact points (scale bar = 4 µm). (**B**) Quantification of the ratio of mitochondria-ER contact surface area to total area of visible hypodermis in wild type, *sel-12(ty11)*, and *mcu-1; sel-12(ty11)* animals (n ≥ 17 animals). (**C**) Mitochondrial-ER contact surface to total surface area ratio in animals treated with either empty vector or *itr-1* RNAi (n ≥ 15 animals). ns p > 0.05, *p < 0.05, **p < 0.01 using one-way ANOVA with Tukey’s multiple comparison test. Comparisons are made to wild type unless otherwise indicated. Error bars indicate mean ± SEM.

First, to determine whether there is evidence of increased inter-organelle contacts between the lysosomes and mitochondria, we quantified the area of contact between these two organelles. We utilized mito-Dendra2, to label mitochondria [51] and NUC-1::mCherry, to label lysosomes [57] in the hypodermis. We found that the mitochondria and lysosomes had an increased ratio of contact surface area to total surface area of the hypodermis in the *sel-12* mutants versus wild type animals (Fig. 5A, B), indicating a greater total area of mitochondria-lysosome contacts within the hypodermis. However, this increase was not dependent on mitochondrial calcium uptake. Indeed, the mitochondria-lysosome contact area of *mcu-1; sel-12* mutants was indistinguishable from *sel-12* mutants and larger than wild type animals (Fig. 5A, B). Additionally, *itr-1* RNAi depletion was also unable to rescue the increase in mitochondria-lysosome contact surface area in *sel-12* mutants (Fig. 5A, C). These data indicate that, despite the impact loss of *mcu-1* or *itr-1* has on lysosome size in *sel-12* mutants, the increased mitochondria-lysosome contacts are not impacted by mitochondria-ER calcium signaling.

**Figure 7.**
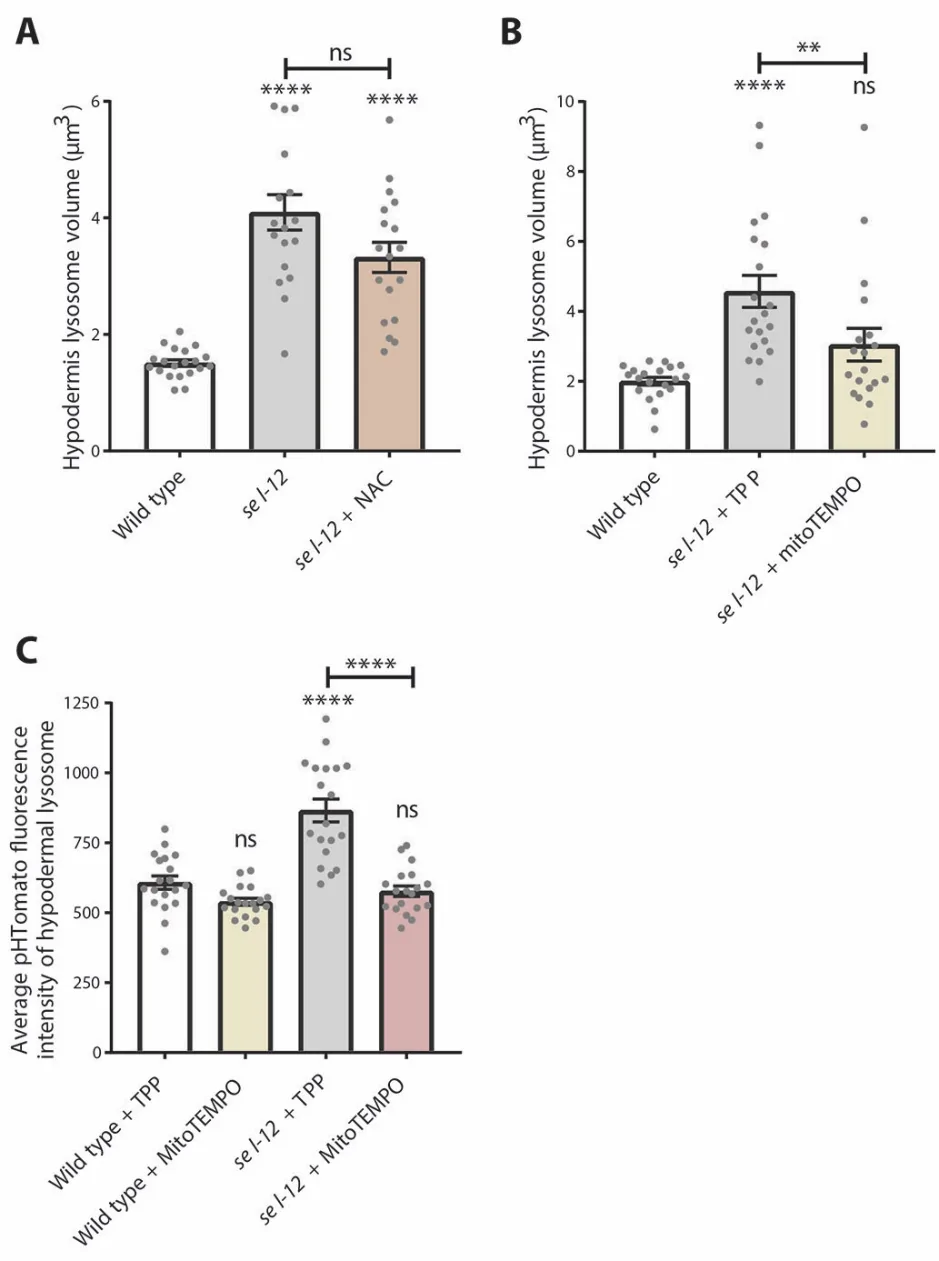
**Lysosome defects are alleviated by mitochondrial-targeted antioxidant treatment**. (**A**) Lysosome volume (*nuc-1*::mCherry) in wild type, *sel-12(ty11)*, and *sel-12(ty11)* animals grown with N-acetylcysteine (NAC) (n ≥ 18 animals). (**B**) Lysosome volume (*nuc-1*::mCherry) in wild type and *sel-12* animals treated with either the antioxidant MitoTEMPO or vehicle (TPP) (n ≥ 19 animals). (**C**) Quantification of the average pHTomato fluorescence intensity per lysosome in animals expressing *nuc-1*::pHTomato controlled by the heat-shock promoter, with increased pHTomato fluorescence intensity indicating increased pH (n ≥ 19 animals). ns p > 0.05, *p < 0.05, ****p < 0.0001. For A and C, one-way ANOVA with Tukey’s multiple comparisons test was used. For B, Kruskal-Wallis test with Dunn’s multiple comparison test was used. Error bars indicate mean ± SEM. Comparisons are made to wild type unless otherwise indicated.

We also examined mitochondria-lysosome colocalization in the TRNs by introducing a TRN-specific marker for mitochondria along with the ubiquitous NUC-1::mCherry lysosome reporter. As in the hypodermis, we found increased lysosome-mitochondria contact area in *sel-12* mutants compared to wild type animals (Fig. 5D, E). However, this increase was not affected by inhibiting mitochondrial calcium uptake. Indeed, introduction of the *mcu-1* null mutation into the *sel-12* mutant background did not show a decrease in TRN mitochondria-lysosome contacts (Fig. 5D, E). Altogether, these data suggest that loss of SEL-12 results in increased contact between the mitochondria and lysosomes, but this is neither influenced by mitochondrial calcium nor ER calcium release, but through an alternate mechanism.

Next, to determine the level of contacts between the ER and the lysosomes, we co-expressed the NUC-1::mCherry lysosomal marker with an ER marker driven under a hypodermal-specific promoter. Similarly, we quantified the contact surface area between the hypodermal ER and lysosomes at a region of interest at the midbody. Like the other inter-organelle interactions, we found increased contact area between the ER and lysosomes in *sel-12* mutants compared to wild type animals (Fig. 6A, B). Markedly, we found that inhibiting mitochondrial calcium uptake using the *mcu-1* null mutation prevented this increase in contact area between the ER and lysosomes in *sel-12* mutants (Fig. 6A, B). Similarly, inhibiting ER calcium release via *itr-1* RNAi also reduced ER-lysosome contact surface area to wild type levels (Fig. 6A, C). These data indicate that ER calcium release as well as mitochondrial calcium uptake influences ER-lysosome contacts, which may alter ER-lysosome communication and impact lysosome function.

### Lysosome defects are alleviated by mitochondrial-targeted antioxidant treatment

To help understand the mechanism connecting mitochondrial calcium dyshomeostasis and lysosome function in *sel-12* mutants, we explored the impact mitochondrial reactive oxygen species (ROS) have on lysosome function. Previously, we demonstrated that *sel-12* mutants show evidence of elevated oxidative stress [13, 18]. In *sel-12* mutants, increased mitochondrial calcium accumulation disrupts mitochondrial activity and results in increased mitochondrial ROS generation. In agreement with this role of calcium, the elevated ROS in *sel-12* mutants is relieved by inhibiting mitochondrial calcium uptake [13, 18]. Increased mitochondrial ROS may increase the misfolded protein load and put a strain upon the lysosome system and may also explain the connection between mitochondrial calcium and lysosomal defects. Indeed, reducing mitochondrial calcium uptake or ROS production was found to improve proteostasis defects in *sel-12* mutants [17]. To determine the role of oxidative stress in aberrant lysosome morphology, we treated *sel-12* mutants with the antioxidant n-acetylcysteine (NAC). However, there was no reduction of lysosome volume with NAC treatment (Fig. 7A). To determine whether specifically targeting mitochondrial ROS would improve lysosome morphology, we treated *sel-12* animals with either MitoTEMPO, which targets the antioxidant piperidine nitroxide (TEMPO) to the mitochondria, or triphenylphosphonium (TPP), the vehicle control. *sel-12* mutants treated with MitoTEMPO showed a significant reduction in lysosome volume compared to *sel-12* mutants treated with TPP (Fig. 7B). Similarly, MitoTEMPO treatment restored *sel-12* mutant lysosome acidity as measured by pHTomato fluorescence intensity (7C). This suggests that mitochondrial ROS, generated by altered ER-to-mitochondria calcium signaling, is responsible for lysosome dysfunction in *sel-12* mutants.

Collectively, these data show that loss of SEL-12 not only promotes interactions between the ER and mitochondria but with the lysosomes as well, which suggests that *sel-12* function is important for regulating interactions between these three types of organelles. These data may also indicate altered signaling between these organelles in *sel-12* mutants, which in turn impacts their function. Furthermore, we have identified aberrant ER-to-mitochondrial calcium signaling in *sel-12* mutants leads to lysosome morphological and acidification defects by increasing mitochondrial ROS, implicating ER-mitochondrial calcium homeostasis as a factor in lysosome health.

## DISCUSSION

Lysosomal function is necessary for maintaining proteostasis, and its dysfunction is associated with Alzheimer’s disease [58, 59]. The phylogenetically conserved protein, presenilin, has been shown to influence lysosome health, but the underlying mechanism is still unclear [10, 14, 20, 22, 24]. In this study, we found that loss of the *C. elegans* presenilin orthologue encoded by the *sel-12* gene disrupts lysosome morphology and function as a result of elevated ER-to-mitochondria calcium signaling. We have identified a novel link between mitochondrial calcium and lysosome health in the context of presenilin function, which may have functional implications into the mechanism underlying fAD. Additionally, this effect is independent of SEL-12’s gamma secretase activity, consistent with other studies showing that presenilin loss impacts lysosome function in a gamma secretase-independent manner [10, 11]. Since the activation of lysosomal proteases requires strict maintenance of acidic pH, the morphological changes and increased pH we observed in *sel-12* null mutants likely undermines lysosome overall function and neuronal fitness.

Presenilin’s best understood role is acting as the proteolytic subunit of gamma secretase. The gamma secretase complex is required for the proteolytic processing of numerous type I transmembrane domain proteins. Recently, it has been demonstrated that the proteolytic activity of presenilin containing gamma secretase is in acidic compartments, including lysosomes [60]. Thus, the lysosomal morphological changes and functional defects we observe in *sel-12* mutants may arise due to the accumulation of unprocessed membrane proteins. However, this notion is unlikely since we did not observe lysosomal morphology defects in *sel-12* mutants that harbor a mutation that abolishes its proteolytic activity (Supplementary Fig. 1D).

The autophagy-lysosome system is particularly important for neurons, which are mostly post-mitotic and rely on this system to clear intracellular waste and maintain proteome integrity. Indeed, neurons are highly sensitive to insults to lysosome function, as this, for example, will compromise autophagy and lead to a buildup of damaged, aggregated material. Morphological changes in the lysosome such as enlargement may indicate dysfunction and the accumulation of undigested materials. Lysosomes display significant variation in size to accommodate the contents delivered to them [61]. Enlarged lysosomes might also result from increased lysosome fusion events [16]. Regardless, indicators of morphological and functional lysosome abnormalities presage neuronal damage. Our findings in *sel-12* mutants agree with those in human fAD models which showed enlarged lysosomes [20, 24] and lysosome acidification defects [10, 21, 22], suggesting this important function of presenilin is highly conserved. Indeed, loss of presenilin in *Dictyostelium* also shows defects in lysosome acidification [14]. Considering that *sel-12* mutants show loss of proteostasis [17], it is likely that lysosome dysfunction contributes to proteostatic collapse. Additionally, since lysosome morphological abnormalities occur in L4 animals (the larval stage before adulthood), whereas neuronal defects are first observed in day 1 adults, this suggests that lysosomal defects precede neuronal dysfunction in *sel-12* mutants [13]. Moreover, our data demonstrate the interdependent nature of organelles, as we show that lysosome dysfunction can stem from dysregulated signaling between the ER and mitochondria. Our findings align with a recent study showing that elevated ER calcium release in an AD model resulted in lysosome alkalization and protein aggregation [62]. Furthermore, another recent study found evidence that elevated ER calcium release in an AD mouse model also inhibits autophagy [63]. However, the mechanism underlying these observations is not clear. Here, we implicate mitochondrial calcium and ER-mitochondrial calcium signaling in defective lysosome acidification.

The effect of mitochondrial calcium on lysosome function is unclear. There are studies that demonstrate that ER-mitochondrial calcium dysregulation impacts mitochondrial ROS production and that lysosome calcium levels are affected by increasing mitochondrial ROS, which is generated as a byproduct of increased calcium-mediated mitochondrial activity [64-66]. Mitochondrial dysfunction can alter lysosomal morphology by generating ROS [67]. Presenilin has been shown to interfere with a lysosomal calcium efflux channel, mucolipin transient receptor potential channel subfamily (TRPML1) [22]. Interestingly, TRPML1’s function has also been shown to be highly ROS-dependent [68]. In general, lysosomes are susceptible to oxidative stress, and lysosome damage by oxidants sensitizes neurons to apoptosis and necrosis [69]. We have previously demonstrated that ROS generation in *sel-12* mutants results from increased mitochondrial calcium uptake, which triggers neurodegeneration [17, 70]. Here, in *sel-12* mutants, we found that mitochondrial ROS plays a role in lysosome enlargement and alkalization (Fig. 7B, C). Furthermore, alkalization of lysosomes, which are calcium harboring stores, has been shown to promote calcium efflux [22]. Thus, the lysosome alkalization we observe in *sel-12* mutants likely leads to further defects in calcium homeostasis.

To better understand the relationship between the ER, mitochondria, and lysosomes and how they might influence each other in absence of SEL-12 function, we quantified inter-organelle contact and found increased contact area between all these organelles in *sel-12* null mutants. This suggests that presenilin has an additional role in preventing inappropriate interactions between organelles, perhaps acting as a gatekeeper for inter-organelle communication and signaling in order to maintain cellular homeostasis. Without SEL-12, these interactions are increased, likely resulting in excessive inter-organelle communication. This altered signaling can in turn have downstream consequences on organelle function and impact cellular fitness. There is previous evidence that null or fAD associated presenilin mutations increase ER-mitochondria contact, upregulate membrane protein function at contact sites, and alter lipid and calcium exchange between the ER and mitochondria [8, 46]. Our data show that loss of SEL-12 function also increases contact between the ER and mitochondria in *C. elegans*, which occurs upstream of elevated mitochondrial calcium, as inhibiting mitochondrial calcium uptake was unable to rescue the increase in ER-mitochondrial contact area (Fig. 4A, B). However, we found that knockdown of the ER inositol 1,4,5-trisphosphate receptors suppressed the increased mitochondrial Ca2+ and ER-mitochondrial contacts observed in *sel-12* mutants (Fig. 4A, C). Importantly, inositol 1,4,5-trisphosphate receptors have been shown to be critical for mediating ER-mitochondrial contact [49-51].

Less is understood about communication between the mitochondria and lysosomes and may be an important understudied relationship influencing neuronal function. Although we found that *sel-12* mutants had increased mitochondrial-lysosome contacts (Fig. 5), the mechanism underlying this aberrant interaction is unclear. There have been studies in other neurodegenerative disease models showing aberrant mitochondria-lysosome contact dynamics that suggest altered communication is involved in disease pathology [71, 72]. Other studies show a close proximity between mitochondria and lysosomes as well as significant crosstalk, especially in regulating calcium homeostasis [73, 74]. Both mitochondria and lysosomes regulate intracellular calcium signaling and are simultaneously influenced by calcium levels [75]. Changes in contact surface area may therefore alter calcium signaling between these two organelles and in turn affect their function. ER-lysosome communication has been even less studied, although there is data indicating that altering ER-lysosome contact affects neuronal health [76, 77]. It is therefore interesting that our findings demonstrate *sel-12* mutants have increased ER-lysosome contact dependent upon ER-to-mitochondrial calcium signaling (Fig. 6), suggesting another avenue by which altered calcium signaling may influence other organelles and ultimately neuronal health.

Altogether, our study demonstrates a role for ER-to-mitochondrial calcium signaling in lysosome dysfunction caused by loss of SEL-12. SEL-12 loss increases ER-mitochondrial contacts, likely promoting ER-to-mitochondrial calcium exchange, which affects the health of not only the mitochondria but the lysosomes, which in turn likely contributes to proteostatic collapse and neurodegeneration in *sel-12* mutants [13, 17]. The influence of mitochondrial calcium on the lysosomes is also conserved in cells isolated from fAD patients, as we found that inhibiting mitochondrial calcium uptake reduced the enlarged lysosomes observed in fibroblasts isolated from patients harboring fAD mutant presenilin. Additionally, our results indicate that SEL-12 may also be important for regulating inter-organelle contacts and communication between not only the ER and mitochondria but with the lysosome as well. Our data therefore show the importance of inter-organelle crosstalk and how intracellular ER-mitochondrial calcium signaling dysregulates lysosome function in *C. elegans,* providing novel insight into the pathology that may underlie Alzheimer’s disease.

## Supplementary Materials

The Supplementary data can be found online at: www.aginganddisease.org/EN/10.14336/AD.2024.0228.

## References

[1] Kaushik S, Cuervo AM (2015). Proteostasis and aging. Nat Med, 21: 1406-15. 10.1038/nm.4001 26646497

[2] Labbadia J, Morimoto RI (2015). The biology of proteostasis in aging and disease. Annu Rev Biochem, 84: 435-64. 10.1146/annurev-biochem-060614-033955 PMC4539002 25784053

[3] Nixon, RA, Yang DS (2011). Autophagy failure in Alzheimer's disease--locating the primary defect. Neurobiol Dis, 43: 38-45. 10.1016/j.nbd.2011.01.021 PMC3096679 21296668

[4] Wolfe DM, Lee JH, Kumar A, Lee S, Orenstein SJ, Nixon RA (2013). Autophagy failure in Alzheimer's disease and the role of defective lysosomal acidification. Eur J Neurosci, 37: 1949-61. 10.1111/ejn.12169 PMC3694736 23773064

[5] Boland B, Kumar A, Lee S, Platt FM, Wegiel J, Yu WH, Nixon RA (2008). Autophagy induction and autophagosome clearance in neurons: relationship to autophagic pathology in Alzheimer's disease. J Neurosci, 28: 6926-37. 10.1523/JNEUROSCI.0800-08.2008 PMC2676733 18596167

[6] Yu WH, Cuervo AM, Kumar A, Peterhoff CM, Schmidt SD, Lee JH, Mohan PS, et al. (2005). Macroautophagy--a novel Beta-amyloid peptide-generating pathway activated in Alzheimer's disease. J Cell Biol, 171: 87-98. 10.1083/jcb.200505082 PMC2171227 16203860

[7] Yang DS, Kumar A, Stavrides P, Peterson J, Peterhoff CM, Pawlik M, et al. (2008). Neuronal apoptosis and autophagy cross talk in aging PS/APP mice, a model of Alzheimer's disease. Am J Pathol, 173: 665-81. 10.2353/ajpath.2008.071176 PMC2527090 18688038

[8] Area-Gomez E, Del Carmen Lara Castillo M, Tambini MD, Guardia-Laguarta C, de Groof AJ, Madra M, et al. (2012). Upregulated function of mitochondria-associated ER membranes in Alzheimer disease. EMBO J, 31: 4106-23. 10.1038/emboj.2012.202 PMC3492725 22892566

[9] Cheung KH, Shineman D, Müller M, Cárdenas C, Mei L, Yang J, et al. (2008). Mechanism of Ca2+ disruption in Alzheimer's disease by presenilin regulation of InsP3 receptor channel gating. Neuron, 58: 871-83. 10.1016/j.neuron.2008.04.015 PMC2495086 18579078

[10] Lee JH, Yu WH, Kumar A, Lee S, Mohan PS, Peterhoff CM, et al. (2010). Lysosomal proteolysis and autophagy require presenilin 1 and are disrupted by Alzheimer-related PS1 mutations. Cell, 141: 1146-58. 10.1016/j.cell.2010.05.008 PMC3647462 20541250

[11] Neely KM, KN Green, LaFerla FM (2011). Presenilin is necessary for efficient proteolysis through the autophagy-lysosome system in a gamma-secretase-independent manner. J Neurosci, 31: 2781-91. 10.1523/JNEUROSCI.5156-10.2010 PMC3064964 21414900

[12] Reddy K, Cusack CL, Nnah IC, Khayati K, Saqcena C, Huynh TB, et al. (2016). Dysregulation of Nutrient Sensing and CLEARance in Presenilin Deficiency. Cell Rep, 14: 2166-2179. 10.1016/j.celrep.2016.02.006 PMC4793148 26923592

[13] Sarasija S, Laboy JT, Ashkavand Z, Bonner J, Tang Y, Norman KR (2018). Presenilin mutations deregulate mitochondrial Ca(2+) homeostasis and metabolic activity causing neurodegeneration in Caenorhabditis elegans. Elife, 7: e33052. 10.7554/eLife.33052 PMC6075864 29989545

[14] Sharma D, Otto G, Warren EC, Beesley P, King JS, Williams RSB (2019). Gamma secretase orthologs are required for lysosomal activity and autophagic degradation in Dictyostelium discoideum, independent of PSEN (presenilin) proteolytic function. Autophagy, 15: 1407-1418. 10.1080/15548627.2019.1586245 PMC6613883 30806144

[15] Tu H, Nelson O, Bezprozvanny A, Wang Z, Lee SF, Hao YH, et al. (2006). Presenilins form ER Ca2+ leak channels, a function disrupted by familial Alzheimer's disease-linked mutations. Cell, 126: 981-93. 10.1016/j.cell.2006.06.059 PMC3241869 16959576

[16] Orr ME, Oddo S (2013). Autophagic/lysosomal dysfunction in Alzheimer's disease. Alzheimers Res Ther, 5: 53. 10.1186/alzrt217 PMC3979020 24171818

[17] Ashkavand Z, Sarasija S, Ryan KC, Laboy JT, Norman KR (2020). Corrupted ER-mitochondrial calcium homeostasis promotes the collapse of proteostasis. Aging Cell, 19: e13065. 10.1111/acel.13065 PMC6974732 31714672

[18] Ryan KC, Ashkavand Z, Sarasija S, Laboy JT, Samarakoon R, Norman KR (2021). Increased Mitochondrial Calcium Uptake and Concomitant Hyperactivity by Presenilin Loss Promotes mTORC1 Signaling to Drive Neurodegeneration. Aging Cell, 19: e13065. 10.1111/acel.13472 PMC8520713 34499406

[19] Neely Kayala KM, Dickinson GD, Minassian A, Walls KC, Green KN, Laferla FM (2012). Presenilin-null cells have altered two-pore calcium channel expression and lysosomal calcium: implications for lysosomal function. Brain Res, 1489: 8-16. 10.1016/j.brainres.2012.10.036 PMC3516298 23103503

[20] Coen K, Flannagan RS, Baron S, Carraro-Lacroix LR, Wang D, Vermeire W, et al. (2012). Lysosomal calcium homeostasis defects, not proton pump defects, cause endo-lysosomal dysfunction in PSEN-deficient cells. J Cell Biol, 198: 23-35. 10.1083/jcb.201201076 PMC3392942 22753898

[21] Coffey EE, Beckel JM, Laties AM, Mitchell CH (2014). Lysosomal alkalization and dysfunction in human fibroblasts with the Alzheimer's disease-linked presenilin 1 A246E mutation can be reversed with cAMP. Neuroscience, 263: 111-24. 10.1016/j.neuroscience.2014.01.001 PMC4028113 24418614

[22] Lee JH, McBrayer MK, Wolfe DM, Haslett LJ, Kumar A, Sato Y, et al. (2015). Presenilin 1 Maintains Lysosomal Ca(2+) Homeostasis via TRPML1 by Regulating vATPase-Mediated Lysosome Acidification. Cell Rep, 12: 1430-44. 10.1016/j.celrep.2015.07.050 PMC4558203 26299959

[23] Nixon RA, Wegiel J, Kumar A, Yu WH, Peterhoff C, Cataldo A, et al. (2005). Extensive involvement of autophagy in Alzheimer disease: an immuno-electron microscopy study. J Neuropathol Exp Neurol, 64: 113-22. 10.1093/jnen/64.2.113 15751225

[24] Hung COY, Livesey FJ (2018). Altered gamma-Secretase Processing of APP Disrupts Lysosome and Autophagosome Function in Monogenic Alzheimer's Disease. Cell Rep, 25: 3647-3660 e2. 10.1016/j.celrep.2018.11.095 PMC6315085 30590039

[25] Lee JH, Yang DS, Goulbourne CN, Im E, Stavrides P, Pensalfini A, et al. (2022). Faulty autolysosome acidification in Alzheimer's disease mouse models induces autophagic build-up of Abeta in neurons, yielding senile plaques. Nat Neurosci, 25: 688-701. 10.1038/s41593-022-01084-8 PMC9174056 35654956

[26] Mello CC, Kramer JM, Stinchcomb D, Ambros V. (1991). Efficient gene transfer in C.elegans: extrachromosomal maintenance and integration of transforming sequences. EMBO J, 10: 3959-70. 10.1002/j.1460-2075.1991.tb04966.x PMC453137 1935914

[27] Timmons L, Fire A (1998). Specific interference by ingested dsRNA. Nature, 395: 854. 10.1038/27579 9804418

[28] Kamath RS, Fraser AG, Dong Y, Poulin G, Durbin R, Gotta M, et al. (2003). Systematic functional analysis of the Caenorhabditis elegans genome using RNAi. Nature, 421: 231-7. 10.1038/nature01278 12529635

[29] Sun Y, Li M, Zhao D, Li X, Yang C, Wang X (2020). Lysosome activity is modulated by multiple longevity pathways and is important for lifespan extension in C. elegans. Elife, 9: e55745. 10.7554/eLife.55745 PMC7274789 32482227

[30] Wang L, Goldwag J, Bouyea M, Barra J, Matteson K, Maharjan N, et al. (2023). Spatial topology of organelle is a new breast cancer cell classifier. iScience, 26: 107229. 10.1016/j.isci.2023.107229 PMC10384275 37519903

[31] Tank EM, Rodgers KE, Kenyon C (2011). Spontaneous age-related neurite branching in Caenorhabditis elegans. J Neurosci, 31: 9279-88. 10.1523/JNEUROSCI.6606-10.2011 PMC3148144 21697377

[32] Toth ML, Melentijevic I, Shah L, Bhatia A, Lu K, Talwar A, et al. (2012). Neurite sprouting and synapse deterioration in the aging Caenorhabditis elegans nervous system. J Neurosci, 32: 8778-90. 10.1523/JNEUROSCI.1494-11.2012 PMC3427745 22745480

[33] Clark SG, Chiu C (2003). C. elegans ZAG-1, a Zn-finger-homeodomain protein, regulates axonal development and neuronal differentiation. Development, 130: 3781-94. 10.1242/dev.00571 12835394

[34] Guo P, Hu T, Zhang J, Jiang S, Wang X (2010). Sequential action of Caenorhabditis elegans Rab GTPases regulates phagolysosome formation during apoptotic cell degradation. Proc Natl Acad Sci U S A, 107: 18016-21. 10.1073/pnas.1008946107 PMC2964220 20921409

[35] Cinar HN, Sweet KL, Hosemann KE, Earley K, Newman AP (2001). The SEL-12 presenilin mediates induction of the Caenorhabditis elegans uterine pi cell fate. Dev Biol, 237: 173-82. 10.1006/dbio.2001.0374 11518514

[36] Levitan D, Greenwald I (1995). Facilitation of lin-12-mediated signalling by sel-12, a Caenorhabditis elegans S182 Alzheimer's disease gene. Nature, 377: 351-4. 10.1038/377351a0 7566091

[37] El Mouridi S, Lecroisey C, Tardy P, Mercier M, Leclercq-Blondel A, Zariohi N, et al.Reliable CRISPR/Cas9 Genome Engineering in Caenorhabditis elegans Using a Single Efficient sgRNA and an Easily Recognizable Phenotype. G3 (Bethesda), 7: 1429-1437. 10.1534/g3.117.040824 PMC5427500 28280211

[38] Li Y, Tsien RW (2012). pHTomato, a red, genetically encoded indicator that enables multiplex interrogation of synaptic activity. Nat Neurosci, 15: 1047-53. 10.1038/nn.3126 PMC3959862 22634730

[39] Chou CC, Vest R, Prado MA, Wilson-Grady J, Paulo JA, Shibuya Y, Moran-Losada P, et al. (2023). Proteostasis and lysosomal quality control deficits in Alzheimer's disease neurons. bioRxiv, 2023.03.27.534444.

[40] Xu S, Chisholm AD (2014). C. elegans epidermal wounding induces a mitochondrial ROS burst that promotes wound repair. Dev Cell, 31: 48-60. 10.1016/j.devcel.2014.08.002 PMC4197410 25313960

[41] Álvarez-Illera P, García-Casas P, Fonteriz RI, Montero M, Alvarez J (2020). Mitochondrial Ca(2+) Dynamics in MCU Knockout C. elegans Worms. Int J Mol Sci, 21: 8622. 10.3390/ijms21228622 PMC7696937 33207633

[42] Lee SY, Hwang DY, Kim YK, Lee JW, Shin IC, Oh KW, et al. (2006). PS2 mutation increases neuronal cell vulnerability to neurotoxicants through activation of caspase-3 by enhancing of ryanodine receptor-mediated calcium release. FASEB J, 20: 151-3. 10.1096/fj.05-4017fje;1 16394273

[43] Green KN, Demuro A, Akbari Y, Hitt BD, Smith IF, Parker I, et al. (2008). SERCA pump activity is physiologically regulated by presenilin and regulates amyloid beta production. J Cell Biol, 181: 1107-16. 10.1083/jcb.200706171 PMC2442205 18591429

[44] Popugaeva E, Pchitskaya E, Bezprozvanny I (2017). Dysregulation of neuronal calcium homeostasis in Alzheimer's disease - A therapeutic opportunity? Biochem Biophys Res Commun, 483: 998-1004. 10.1016/j.bbrc.2016.09.053 PMC5303663 27641664

[45] Hedskog L, Pinho CM, Filadi R, Rönnbäck A, Hertwig L, Wiehager B, et al. (2013). Modulation of the endoplasmic reticulum-mitochondria interface in Alzheimer's disease and related models. Proc Natl Acad Sci U S A, 110: 7916-21. 10.1073/pnas.1300677110 PMC3651455 23620518

[46] Zampese E, et al., Presenilin 2 modulates endoplasmic reticulum (ER)-mitochondria interactions and Ca2+ cross-talk. Proc Natl Acad Sci U S A, 2011. 108(7): p. 2777-82. 10.1073/pnas.1100735108 PMC3041131 21285369

[47] Area-Gomez E, de Groof AJ, Boldogh I, Bird TD, Gibson GE, Koehler CM, et al. (2009). Presenilins are enriched in endoplasmic reticulum membranes associated with mitochondria. Am J Pathol, 175: 1810-6. 10.2353/ajpath.2009.090219 PMC2774047 19834068

[48] Area-Gomez E, Schon EA (2017). On the Pathogenesis of Alzheimer's Disease: The MAM Hypothesis. FASEB J, 31: 864-867. 10.1096/fj.201601309 PMC6191063 28246299

[49] Xie L, Gao S, Alcaire SM, Aoyagi K, Wang Y, Griffin JK, et al. (2013). NLF-1 delivers a sodium leak chennel to regulate neuronal excitability and modulate rhythmic locomotion. Neuron, 77: 1069-82. 10.1016/j.neuron.2013.01.018 23522043

[50] Levitan D, Greenwald I (1998). Effects of SEL-12 presenilin on LIN-12 localization and function in Caenorhabditis elegans. Development, 125: 3599-606. 10.1242/dev.125.18.3599 9716525

[51] Xu S, Wang Z, Kim KW, Jin Y, Chisholm AD (2016). Targeted Mutagenesis of Duplicated Genes in Caenorhabditis elegans Using CRISPR-Cas9. J Genet Genomics, 43: 103-6. 10.1016/j.jgg.2015.11.004 PMC5291165 26924693

[52] Szabadkai G, Bianchi K, Várnai P, De Stefani D, Wieckowski MR, Cavagna D, et al. (2006). Chaperone-mediated coupling of endoplasmic reticulum and mitochondrial Ca2+ channels. J Cell Biol, 175: 901-11. 10.1083/jcb.200608073 PMC2064700 17178908

[53] Csordas G, Weaver D, Hajnoczky G (2018). Endoplasmic Reticulum-Mitochondrial Contactology: Structure and Signaling Functions. Trends Cell Biol, 28: 523-540. 10.1016/j.tcb.2018.02.009 PMC6005738 29588129

[54] Bartok A, Weaver D, Golenár T, Nichtova Z, Katona M, Bánsághi S, et al. (2019). IP(3) receptor isoforms differently regulate ER-mitochondrial contacts and local calcium transfer. Nat Commun, 10: 3726. 10.1038/s41467-019-11646-3 PMC6700175 31427578

[55] Pasternak SH, Bagshaw RD, Guiral M, Zhang S, Ackerley CA, Pak BJ, et al. (2003). Presenilin-1, nicastrin, amyloid precursor protein, and gamma-secretase activity are co-localized in the lysosomal membrane. J Biol Chem, 278: 26687-94. 10.1074/jbc.m304009200 12736250

[56] Sannerud R, Esselens C, Ejsmont P, Mattera R, Rochin L, Tharkeshwar AK, et al. (2016). Restricted Location of PSEN2/gamma-Secretase Determines Substrate Specificity and Generates an Intracellular Abeta Pool. Cell, 166: 193-208. 10.1016/j.cell.2016.05.020 PMC7439524 27293189

[57] Li Y, Chen B, Zou W, Wang X, Wu Y, Zhao D, et al.The lysosomal membrane protein SCAV-3 maintains lysosome integrity and adult longevity. J Cell Biol, 215: 167-185. 10.1083/jcb.201602090 PMC5084646 27810910

[58] Bonam SR, Wang F, Muller S (2019). Lysosomes as a therapeutic target. Nat Rev Drug Discov, 18: 923-948. 10.1038/s41573-019-0036-1 PMC7097195 31477883

[59] Zhang L, Sheng R, Qin Z (2009). The lysosome and neurodegenerative diseases. Acta Biochim Biophys Sin (Shanghai), 41: 437-45. 10.1093/abbs/gmp031 19499146

[60] Maesako M, Houser MCQ, Turchyna Y, Wolfe MS, Berezovska O (2022). Presenilin/gamma-Secretase Activity Is Located in Acidic Compartments of Live Neurons. J Neurosci, 42: 145-154. 10.1523/JNEUROSCI.1698-21.2021 PMC8741161 34810230

[61] de Araujo MEG, Liebscher G, Hess MW, Huber LA (2020). Lysosomal size matters. Traffic, 21: 60-75. 10.1111/tra.12714 PMC6972631 31808235

[62] Mustaly-Kalimi S, Gallegos W, Marr RA, Gilman-Sachs A, Peterson DA, Sekler I, et al. (2022). Protein mishandling and impaired lysosomal proteolysis generated through clacium dysregulation in Alzheimer's disease. Proc Natl Acad Sci U S A, 119: e2211999119. 10.1073/pnas.2211999119 PMC9894236 36442130

[63] Zhang H, Knight C, Chen SRW, Bezprozvanny I (2023). A Gating Mutation in Ryanodine Receptor Type 2 Rescues Phenotypes of Alzheimer's Disease Mouse Models by Upregulating Neuronal Autophagy. J Neurosci, 43: 1441-1454. 10.1523/JNEUROSCI.1820-22.2022 PMC9987572 36627208

[64] Booth DM, Enyedi B, Geiszt M, Várnai P, Hajnóczky G (2016). Redox Nanodomains Are Induced by and Control Calcium Signaling at the ER-Mitochondrial Interface. Mol Cell, 63: 240-248. 10.1016/j.molcel.2016.05.040 PMC4998968 27397688

[65] Song SB, Hwang ES (2020). High Levels of ROS Impair Lysosomal Acidity and Autophagy Flux in Glucose-Deprived Fibroblasts by Activating ATM and Erk Pathways. Biomolecules, 10: 761. 10.3390/biom10050761 PMC7277562 32414146

[66] Yuan Y, Chen Y, Peng T, Li L, Zhu W, Liu F, et al. (2019). Mitochondrial ROS-induced lysosomal dysfunction impairs autophagic flux and contributes to M1 macrophage polarization in a diabetic condition. Clin Sci (Lond), 133: 1759-1777. 10.1042/CS20190672 31383716

[67] Demers-Lamarche J, Guillebaud G, Tlili M, Todkar K, Bélanger N, Grondin M, et al. (2016). Loss of Mitochondrial Function Impairs Lysosomes. J Biol Chem, 291: 10263-76. 10.1074/jbc.M115.695825 PMC4858975 26987902

[68] Zhang X, Cheng X, Yu L, Yang J, Calvo R, Patnaik S, et al. (2016). MCOLN1 is a ROS sensor in lysosomes that regulates autophagy. Nat Commun, 7: 12109. 10.1038/ncomms12109 PMC4931332 27357649

[69] Pivtoraiko VN, Stone SL, Roth KA, Shacka JJ (2009). Oxidative stress and autophagy in the regulation of lysosome-dependent neuron death. Antioxid Redox Signal, 11: 481-96. 10.1089/ars.2008.2263 PMC2933567 18764739

[70] Ryan KC, Laboy JT, Norman KR (2022). Deregulation of Mitochondrial Calcium Handling Due to Presenilin Loss Disrupts Redox Homeostasis and Promotes Neuronal Dysfunction. Antioxidants (Basel), 11: 1642. 10.3390/antiox11091642 PMC9495597 36139715

[71] Höglinger D, Burgoyne T, Sanchez-Heras E, Hartwig P, Colaco A, Newton J, et al. (2019). NPC1 regulates ER contacts with endocytic organelles to mediate cholesterol egress. Nat Commun, 10: 4276. 10.1038/s41467-019-12152-2 PMC6753064 31537798

[72] Cisneros J, Belton TB, Shum GC, Molakal CG, Wong YC (2022). Mitochondria-lysosome contact site dynamics and misregulation in neurodegenerative diseases. Trends Neurosci, 45: 312-322. 10.1016/j.tins.2022.01.005 PMC8930467 35249745

[73] Wong YC, Kim S, Peng W, Krainc D. (2019). Regulation and Function of Mitochondria-Lysosome Membrane Contact Sites in Cellular Homeostasis. Trends Cell Biol, 29: 500-513. 10.1016/j.tcb.2019.02.004 PMC8475646 30898429

[74] Peng W, Wong YC, Krainc D (2020). Mitochondria-lysosome contacts regulate mitochondrial Ca(2+) dynamics via lysosomal TRPML1. Proc Natl Acad Sci U S A,. 117: 19266-19275. 10.1073/pnas.2003236117 PMC7430993 32703809

[75] Feng X, Yang J (2016). Lysosomal Calcium in Neurodegeneration. Messenger (Los Angel), 5: 56-66. 10.1166/msr.2016.1055 PMC5659362 29082116

[76] Özkan N, Koppers M, van Soest I, van Harten A, Jurriens D, Liv N, et al. (2021). ER - lysosome contacts at a pre-axonal region regulate axonal lysosome availability. Nat Commun, 12: 4493. 10.1038/s41467-021-24713-5 PMC8302662 34301956

[77] Lu M, van Tartwijk FW, Lin JQ, Nijenhuis W, Parutto P, Fantham M, et al. (2020). The structure and global distribution of the endoplasmic reticulum network are actively regulated by lysosomes. Sci Adv, 6: eabc7209. 10.1126/sciadv.abc7209 PMC7744115 33328230

