# Supplementary material for: C. elegans Presenilin Mediates Inter-Organelle Contacts and Communication that Is Required for Lysosome Activity
Source: Aging Dis. 2024 Feb 28;16(5):3022–39. doi: 10.14336/AD.2024.0228 (PMC12339110; doi:10.14336/AD.2024.0228)
Supplement: Supplementary file 1 — The Supplementary data can be found online at: www.aginganddisease.org/EN/10.14336/AD.2024.0228. [file AD-16-5-3022-s.pdf]

SUPPLEMENTARY DATA

C. elegans Presenilin Mediates Inter-Organelle Contacts and Communication that Is Required for Lysosome Activity

Kerry C. Ryan, Zahra Ashkavand, Jocelyn T. Laboy, Ling Wang, Margarida Barroso, Kenneth R. Norman

© 2024. Ryan KC et al. Published online at http://www.aginganddisease.org/EN/10.14336/AD.2024.0228

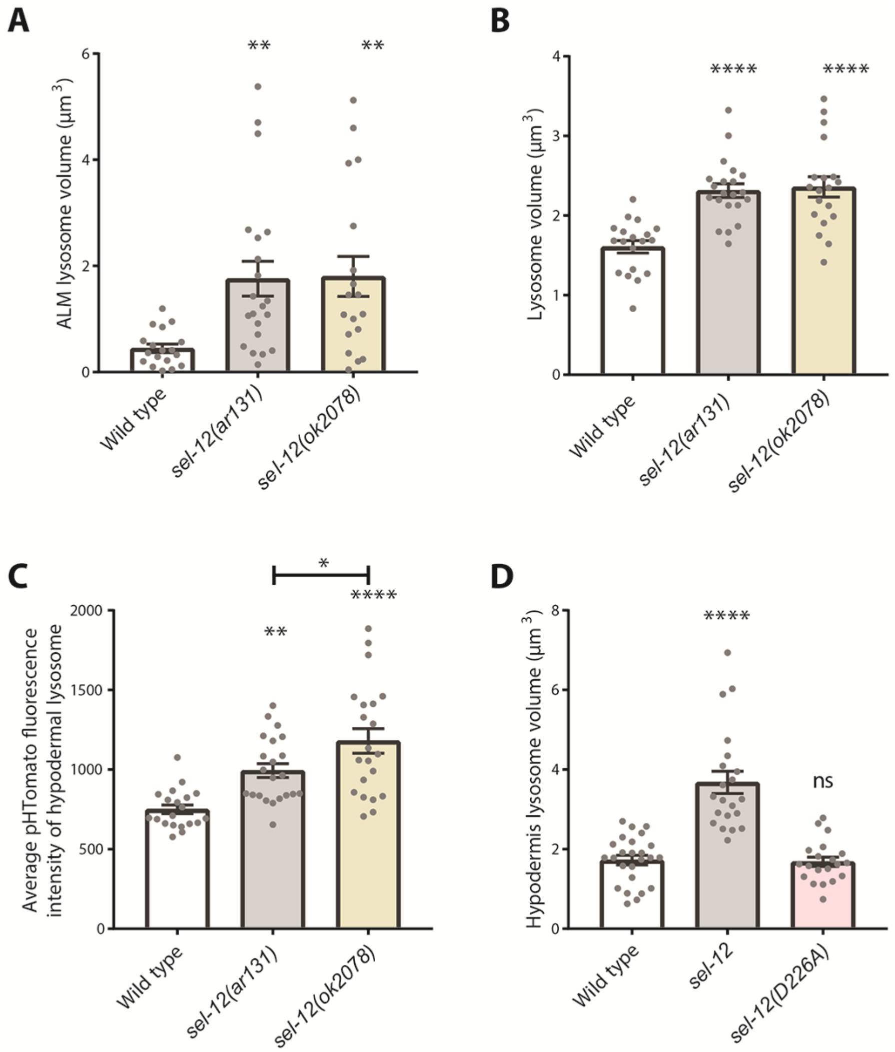

Supplementary Figure 1. Lysosome acidification and morphological defects in *sel-12* mutants are independent of gamma-secretase activity. Related to Figures 1 and 2. (A) Quantification of average lysosome volume within the ALM TRN soma of wild type, *sel-12(ar131),* and *sel-12(ok2078)* animals co-expressing *nuc-1*::mCherry to mark lysosomes and *mec-4p*::GFP to mark the TRNs (n ≥ 20 animals). (B) Quantification of average hypodermal lysosome volume images in animals expressing *nuc-1*::mCherry as a marker for the lysosomal lumen (n ≥ 19 animals). (C) Quantification of the average pHTomato fluorescence intensity per lysosome in animals expressing *nuc-1*::pHTomato controlled by the heat-shock promoter, with increased pHTomato fluorescence intensity indicating increased pH (n ≥ 20 animals). (D) Lysosome volume (*nuc-1*::mCherry) in wild type, null *sel-12(ty11)*, and *sel-12*(D226A), which carry a point mutation in a residue necessary for gamma secretase activity (n ≥ 20 animals). *p < 0.05, ****p < 0.0001 using Kruskal-Wallis with Dunn's multiple comparison test. Comparisons are made to wild type unless otherwise indicated. Error bars indicate mean ± SEM.

© 2024. Ryan KC et al. Published online at http://www.aginganddisease.org/EN/10.14336/AD.2024.0228

SUPPLEMENTARY DATA

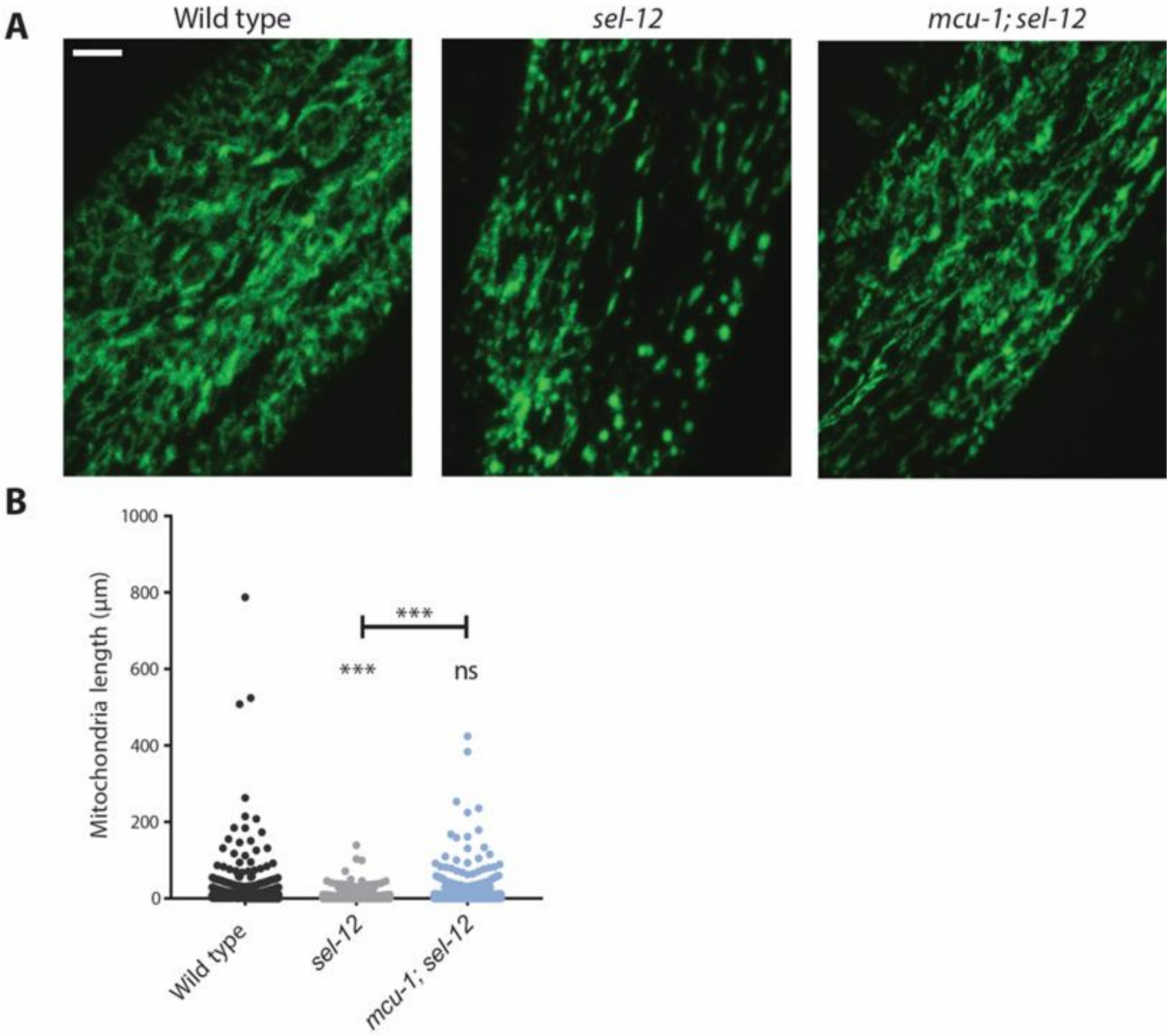

Supplementary Figure 2. ***sel-12(ty11)*** **mutants show fragmented mitochondria in the hypodermis. Related to Figure 2.** (A) Representative images of hypodermal 2xMLS::GCaMP6f expression (scale bar = 20 µm) and (B) quantification of hypodermal mitochondrial length. ns $p > 0.05$, ***$p < 0.001$ using chi-squared test. n = 20 animals. All comparisons are made to wild type animals unless indicated. Error bars indicate mean ± SEM.

© 2024. Ryan KC et al. Published online at http://www.aginganddisease.org/EN/10.14336/AD.2024.0228

SUPPLEMENTARY DATA

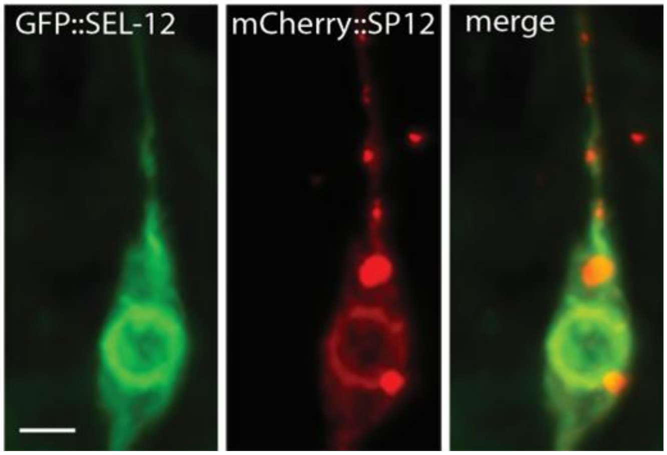

Supplementary Figure 3. SEL-12 localizes to the ER. Related to Figure 4. Confocal image of ALM soma in animal co-expressing a functional SEL-12 GFP fusion protein (*sel-12p::sel-12*::GFP) and pan-neuronal ER reporter (*rgef-1p*::mCherry::SP12) (scale bar = 5 μm).

© 2024. Ryan KC et al. Published online at http://www.aginganddisease.org/EN/10.14336/AD.2024.0228
